# A Bioinspired Immunostimulatory System for Inducing Powerful Antitumor Immune Function by Directly Causing Plasma Membrane Rupture

**DOI:** 10.1002/advs.202305934

**Published:** 2024-03-14

**Authors:** Xiaoqu Hu, Hao Yin, Danli Xie, Tanzhou Chen, Yida Li, Hanqian Zeng, Mingdong Lu, Qinyang Wang

**Affiliations:** ^1^ Department of Radiation and Medical Oncology Wenzhou Key Laboratory of Basic Science and Translational Research of Radiation Oncology Zhejiang Engineering Research Center for Innovation and Application of Intelligent Radiotherapy Technology The Second Affiliated Hospital and Yuying Children’s Hospital of Wenzhou Medical University Wenzhou Zhejiang 325027 P. R. China; ^2^ Institute for Advanced Research Wenzhou Medical University Wenzhou Zhejiang 325027 P. R. China; ^3^ The First Affiliated Hospital of Wenzhou Medical University Wenzhou Medical University Wenzhou Zhejiang 325027 P. R. China

**Keywords:** antitumor immune function, artificial cell membrane disruptor, bioinspired system, plasma membrane rupture

## Abstract

The Gasdermin protein is a membrane disruptor that can mediate immunogenic pyroptosis and elicit anti‐tumor immune function. However, cancer cells downregulate Gasdermin and develop membrane repair mechanisms to resist pyroptosis. Therefore, an artificial membrane disruptor (AMD) that can directly mediate membrane rupture in pyroptosis‐deficient cells and induce antitumor immune responses in a controllable manner will be valuable in preclinical and clinical research. A micron‐scale Ce6‐based AMD that can directly induce plasma membrane rupture (PMR) in gasdermin‐deficient tumor cells is established. Micron‐scale AMDs localize Ce6 specifically to the plasma membrane without labeling other organelles. Compared to free Ce6 molecules, the use of AMDs results in a higher degree of specificity for the plasma membrane. Due to this specificity, AMDs mediate fast and irreversible PMR under 660 nm red light. Furthermore, the AMDs are capable of inducing programmed cell death and lytic cell death in a catalytic manner, demonstrating that the amount of Ce6 used by AMDs is only one‐fifth of that used by Ce6 alone when inducing 80% of cancer cell death. In vivo, the AMDs show specificity for tumor targeting and penetration, suggesting that light‐driven programmed cell death is specific to tumors. AMDs are applied to antitumor therapy in gasdermin‐deficient tumors, resulting in efficient tumor elimination with minimal damage to major organs when combined with anti‐PD‐1 therapy. Tumor regression is correlated with PMR‐mediated inflammation and T‐cell‐based immune responses. This study provides new insights for designing bioinspired membrane disruptors for PMR and mediating anti‐tumor immunotherapy. Additionally, AMD is a dependable tool for examining the immunogenicity of PMR both in vitro and in vivo.

## Graphical abstract

A bioinspired system mediates oncolysis efficiently by inducing plasma membrane rupture (PMR) in a catalytic and controllable manner. It is noteworthy that the plasma membrane rupture successfully triggers antitumor immune function in pyroptosis‐deficient tumors.

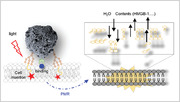

## 1 Introduction

Pyroptosis is a form of immunogenic cell death that is characterized by plasma membrane rupture (PMR).^[^ ^1^, ^2^, ^3^ ^]^ Gasdermin protein, which disrupts the cell membrane, has been identified as the final executor in pyroptosis.^[^ ^4^, ^5^, ^6^ ^]^ Pyroptosis typically results in cell swelling, blistering, and the secretion of pro‐inflammatory cytokines such as IL‐1 and HMGB1.^[^ ^5^, ^6^ ^]^ Recent studies have demonstrated that pyroptosis can enhance antitumor immunity, indicating that gasdermin‐mediated pyroptotic cell death has the potential to improve the efficiency of antitumor immunotherapy.^[^ ^1^, ^2^, ^3^, ^7^ ^]^ However, the expression of gasdermin is often downregulated in human cancer cells, making it impractical to design activators that directly induce gasdermin‐mediated pyroptotic cell death.^[^ ^5^, ^8^ ^]^ Although some nanocarriers have successfully delivered or upregulated gasdermin, the overall efficiency remains low due to the endosome escape problem.^[^ ^1^, ^3^, ^9^ ^]^ Furthermore, gasdermin is highly expressed in normal tissues and cells, particularly immune cells, and non‐specific pyroptosis can lead to cytokine release syndrome (CRS).^[^ ^5^, ^10^, ^11^ ^]^ The challenges of developing strategies based on gasdermin activation in anti‐tumor immunotherapy are evident.^[^ ^8^ ^]^ However, the impressive and inspiring anti‐tumor immune function induced by PMR during pyroptosis highlights the potential of an artificial cell membrane disruptor (AMD) capable of directly mediating PMR and immunogenic cell death for antitumor immunotherapy, particularly for pyroptosis‐deficient tumors.

**Figure 1:**
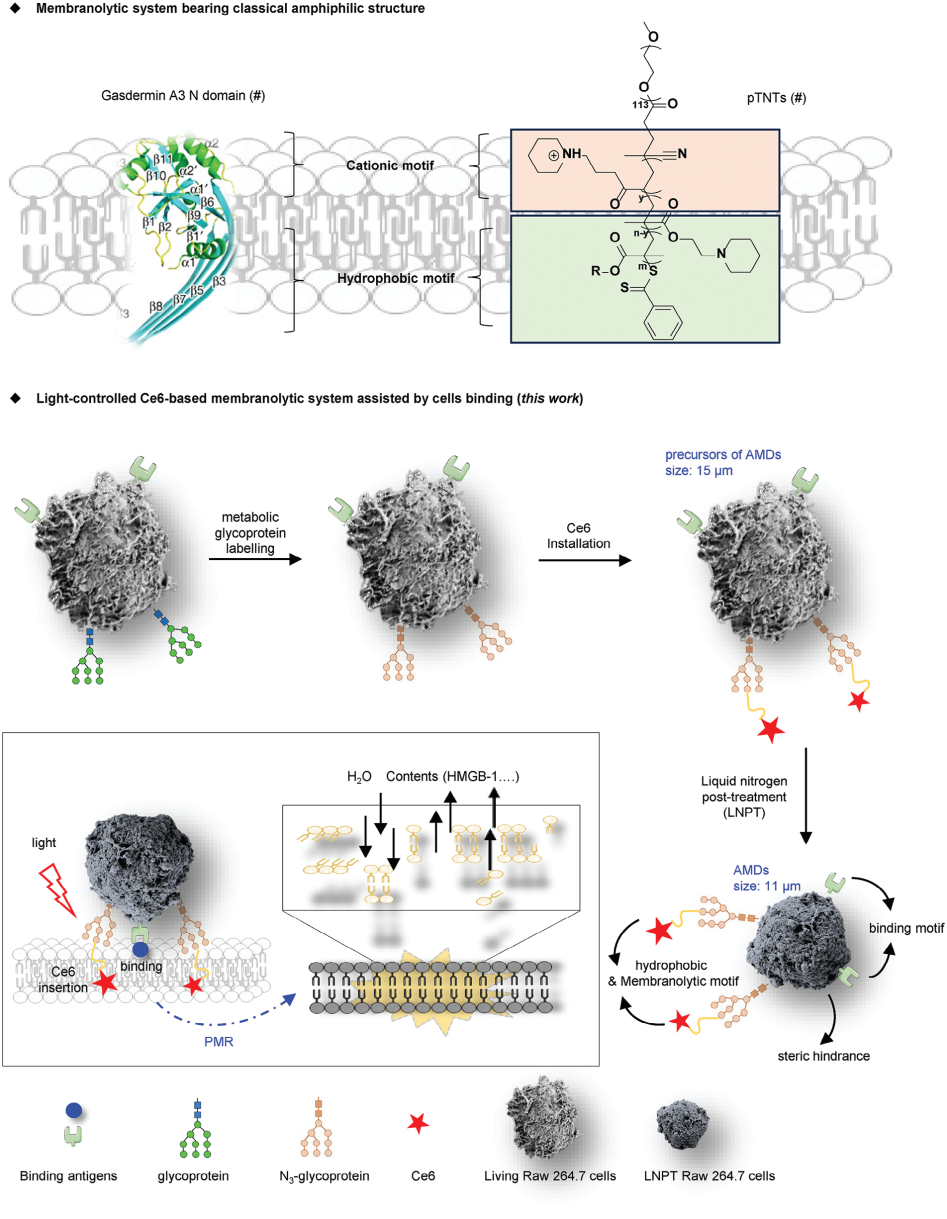
An illustration of the structure of AMDs. The membranolytic systems with amphiphilic structures, such as gasdermin proteins and artificial membranolytic small molecules, use a cationic motif to bind the cell membrane and a hydrophobic motif to insert into the cell membrane to lyse the cell. In the Ce6‐based membranolytic system controlled by light, binding is mediated by antigen recognition. When the antigen is recognized, the hydrophobic Ce6 motif inserts into the cell membrane, generating ROS under light and directing PMR. Ce6 is unable to penetrate the cell membrane due to its large steric hindrance and thus localizes in the cell membrane.

Recently, small molecule‐based membranolytic systems have been established to mediate PMR, for example, a system called pTNTs that can respond to pH.^[^ ^12^, ^13^ ^]^ Similar to the structure of gasdermin,^[^ ^4^, ^5^, ^6^ ^]^ pTNTs have a classical amphiphilic structure that includes cationic and hydrophobic moieties (**Figure** **1**).^[^ ^13^ ^]^ The cationic components bind to the negatively charged cell membrane through electrostatic attraction, while the hydrophobic regions insert into phospholipid bilayers, inducing a membranolytic effect.^[^ ^13^ ^]^ However, their complex structure, high dosage, poor tumor targeting, and unclear immune responses have limited their application in anti‐tumor immunotherapy. Furthermore, the pH‐sensitive molecules' tumor specificity was not high enough to distinguish between subtle pH differences in tumor tissues/cells and normal tissues/cells, which could lead to CRS.^[^ ^14^, ^15^ ^]^ Designing an AMD that can induce anti‐tumor immune function in a controlled, tumor‐specific, safe, and catalytic manner has been a persistent challenge.

Chlorine e6 (Ce6) is a second‐generation photosensitizer approved by the FDA. Its high capacity to generate reactive oxygen species (ROS) gives it the potential to mediate PMR through light‐driven lipid peroxidation.^[^ ^16^, ^17^, ^18^ ^]^ However, due to its high lipophilicity, Ce6 is distributed in all membranous organelles and can induce various types of cell death under light, including non‐immunogenic apoptosis.^[^ ^16^, ^17^, ^18^ ^]^ Furthermore, Ce6's hydrophobicity has several drawbacks, including inadequate biodistribution, high dosage, phototoxicity, and rapid clearance from circulation.^[^ ^17^ ^]^ Recently, Ce6‐based nanoparticles have been shown to improve tumor targeting and facilitate anti‐tumor therapy.^[^ ^16^, ^17^, ^18^ ^]^ These nanoparticles activate the cell death pathway through light‐driven generation of ROS upon entry into cancer cells.^[^ ^16^, ^17^, ^18^ ^]^ However, in tumor cells lacking intrinsic conditions for immunogenic cell death (e.g., pyroptosis‐deficient cells), these ROS in the cytoplasm often activate apoptosis,^[^ ^11^ ^]^ which is characterized by cell membrane integrity and immune silence, reducing the likelihood that anti‐tumor immune functions will be activated; moreover, an increasing number of studies have shown that nanoparticles are readily cleared in vivo by phagocytic cells (e.g., macrophages), reducing their efficacy.^[^ ^19^, ^20^ ^]^ Therefore, an AMD that specifically localizes Ce6 to the cell membrane and triggers PMR under photocontrol is still lacking; furthermore, it is not known whether light‐driven Ce6‐based AMD‐mediated PMR triggers an anti‐tumor immune response.

Here, we established a readily prepared Ce6‐based light‐activated AMD with catalytic killing and specific oncolysis ability to explore PMR‐mediated antitumor immune function in pyroptosis‐deficient tumors. Unlike the reported amphiphilic structure of membranolytic systems (e.g., proteins and small molecules),^[^ ^6^, ^12^, ^13^ ^]^ our AMDs bound tumor cells by taking advantage of antigen‐directed recognition, facilitating the precise insertion of the lipophilic Ce6 regions into the target plasma membrane (Figure 1). Once bound, the large steric hindrance provided by the macrophage carrier prevented Ce6 from labeling other organelles (Figure 1). Specifically, our AMDs were derived from the Ce6‐based chemically engineered macrophage system based on our recently reported liquid nitrogen freezing method (Figure 1).^[^ ^7^, ^8^ ^]^ The chemical engineering approach was a bioorthogonal reaction based on mannose metabolism,^[^ ^21^, ^22^ ^]^ which installed the Ce6‐functionalized glycoprotein arms on the surface of the AMDs. Under 660 nm light irradiation, the irreversible light‐driven lipid peroxidation successfully mediated PMR‐induced cell death in gasdermin‐deficient tumor cells (Figure 1). Without light, there was no cell death and no cytokine release. Furthermore, the AMDs themselves were non‐toxic and would not cause inflammation in vivo. Notably, the AMDs exhibited anti‐corrosion and catalytic killing ability under light, such that the Ce6 required to mediate 80% cell death (IC80) is one‐thirtieth and one‐seventieth of that required for Ce6 alone and cisplatin treatment, respectively. Since our AMDs exhibited tumor selectivity, the red light mediated PMR became tumor‐specific. We applied the AMDs to gasdermin‐deficient tumor‐bearing mice, which showed a significant immune response in the tumors. To our delight, synergy with anti‐PD‐1 therapy resulted in potent antitumor immunity and tumor regression. Our study provides new insights into the design of the artificial membrane disruptor for PMR and the induction of immunogenic cell death. Moreover, the AMD is a reliable tool for investigating the immunogenicity of cell death characterized by PMR.

## 2 Results

### 2.1 Design and Generation of AMDs

**Figure 2:**
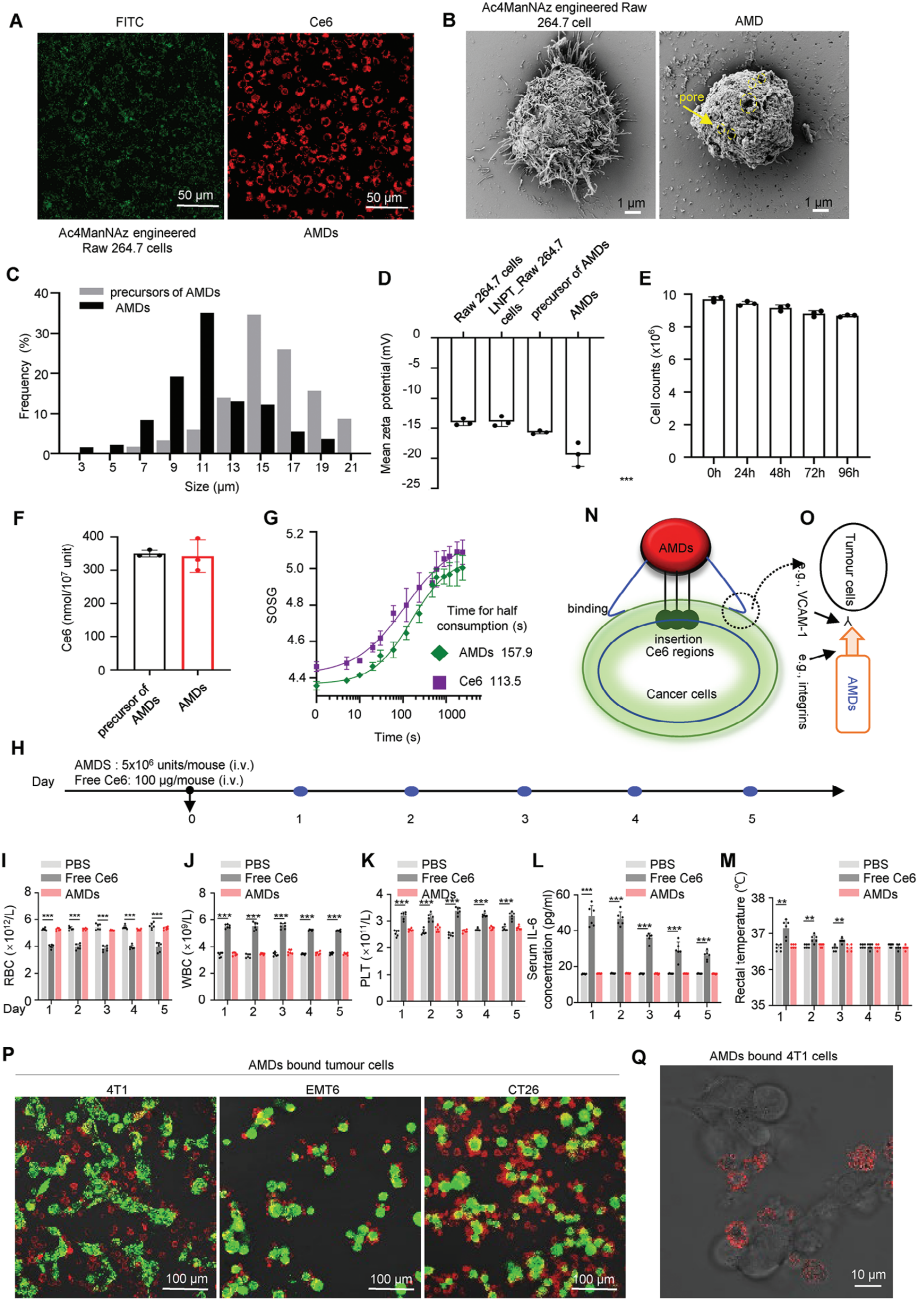
Characterization of AMDs. A) Confocal images of Ac4ManNAz‐engineered living Raw 264.7 cells and AMDs. The Ac4ManNAz engineered Raw 264.7 cells were labelled by FITC‐DBCO. The AMDs were imaged using the fluorescence of Ce6. B) Representative SEM images of precursors of AMDs (left) and AMDs (right). Scale bar, 1 µm. Yellow circles and arrows indicate pores in AMDs. C) Particle size distribution of AMDs and precursors of AMDs. D) The zeta potential of Raw 264.7 cells, LNT‐Raw 264.7 cells (liquid nitrogen treated Raw 264.7 cells), precursors of AMDs, and AMDs. Data are shown as mean ± s.d. (*n* = 3, independent experiments). E) The stability of AMDs in phosphate‐buffered saline (37 °C). The variation in quantity of AMDs based on particle size was determined using a cell counter assay (10^7^ units per tube). Data are shown as mean ± s.d. (*n* = 3, independent experiments). F) The Ce6 concentration of precursor of AMDs (10^7^ cells) and AMDs (10^7^ units). Data are shown as mean ± s.d. (*n* = 3, independent experiments). G) Light (660 nm, 1.0 W cm^−2^) controlled singlet oxygen generation by AMDs and Ce6 alone. H) The safety testing of AMDs and Ce6 in vivo. On day 1, 2, 3, 4, and 5 after intravenously injection of AMDs (5 x 10^6^ units per mouse) and Ce6 (100 µg per mouse), respectively. The blood was gained from the eyeballs of mice. J,K) The RBC I), WBC (J) and PLT (K) of mice was measured at the indicated time. L) Serum IL‐6 concentration of mice. M) The rectal temperature of mice was measured at the indicated time. All measurements (*n* = 3 mice) are biologically independent. Data are shown as mean ± s.d., ^***^, *p* < 0.001, ^**^, *p* < 0.01. N) A schematics of how the AMDs inserted their Ce6 regions to the binding cells membrane. O) A schematic of the AMDs recognizing tumor cells by antigen coordination (e.g., VCAM‐1 and integrins). P) Representative confocal images of the binding between Dil‐labelled AMDs and Calcein‐AM‐labelled tumor cells (4T1, EMT6, and CT26). Q) The AMDs bound 4T1 cells were imaged by LSM representatively. The red fluorescence indicated the Ce6 on the AMDs. Scale bars, 10 µm.

As mentioned above, macrophages were converted into AMDs by the “click reaction” and liquid nitrogen post‐treatment (LNPT).^[^ ^7^, ^8^, ^21^, ^22^ ^]^ The typical procedure is described below: Ac4ManNAz, an azido‐containing metabolic glycoprotein labeling reagent, was used to modify the Raw 264.7 cells. After careful screening and testing, the Ac4ManNAz‐modified living Raw 264.7 cells were successfully produced (Figure 1; **Figure** **2**A). Then, the red light‐activatable photosensors Ce6 were attached to the azided glycoprotein arms by Cu(I)‐catalyzed azide‐alkyne cycloaddition to obtain the precursors of AMDs (Figure 1; Figure S1A, Supporting Information). Finally, the AMDs were obtained by direct treatment of the above precursors with liquid nitrogen. Obviously, all components of the AMDs were biodegradable (Figure 1), indicating that the AMDs have high potential in preclinical and clinical research.

In terms of morphology, the AMDs were 11 µm porous spheres with high dispersion (Figure 2A–C). Compared to the precursors of AMDs, the AMDs showed relatively smaller particle size and lower zeta potential, which may be due to the crumpling and membrane permeability caused by liquid nitrogen treatment (Figure 2C,D). In terms of stability, AMDs showed almost no morphological changes in 96‐h imaging and particle size analysis, indicating the high stability of AMDs (Figure 2E; Figure S1B, Supporting Information). In terms of light‐driven ROS generation capacity, although the surface morphology of AMDs became porous due to liquid nitrogen treatment (Figure 2B), fluorescence imaging and quantitative analysis showed that the Ce6‐bound glycoproteins did not significantly decrease (Figure 2F; Figure S1C, Supporting Information). Thus, when the AMDs were exposed to red light, a significant amount of light‐driven ROS production was detected (Figure 2G; Figure S1D,E, Supporting Information). AMDs were also exposed to continuous red light for 20 minutes and analyzed for changes in morphology and particle size. The images showed that AMDs did not disintegrate during light treatment, while their particle size remained stable, indicating that AMDs are capable of resisting light and ROS corrosion (Figure S1F,G, Supporting Information). In terms of safety, light‐driven ROS generation induced proinflammatory responses and carrier cell death in AMD precursors (Figure S2A–C, Supporting Information). However, no inflammatory molecules were found in AMDs with or without light (Figure S2A, Supporting Information). Moreover, when we intravenously administered the AMDs or Ce6 alone to mice in a five‐day follow‐up analysis, the AMDs‐treated mice showed no significant changes in blood parameters, body temperature, and IL‐6, while the Ce6 alone treated mice showed significant abnormalities in blood parameters and IL‐6, indicating that the AMDs showed higher safety than Ce6 (Figure 2H–M).

### 2.2 The AMDs‐Mediated PMR Under Light

**Figure 3:**
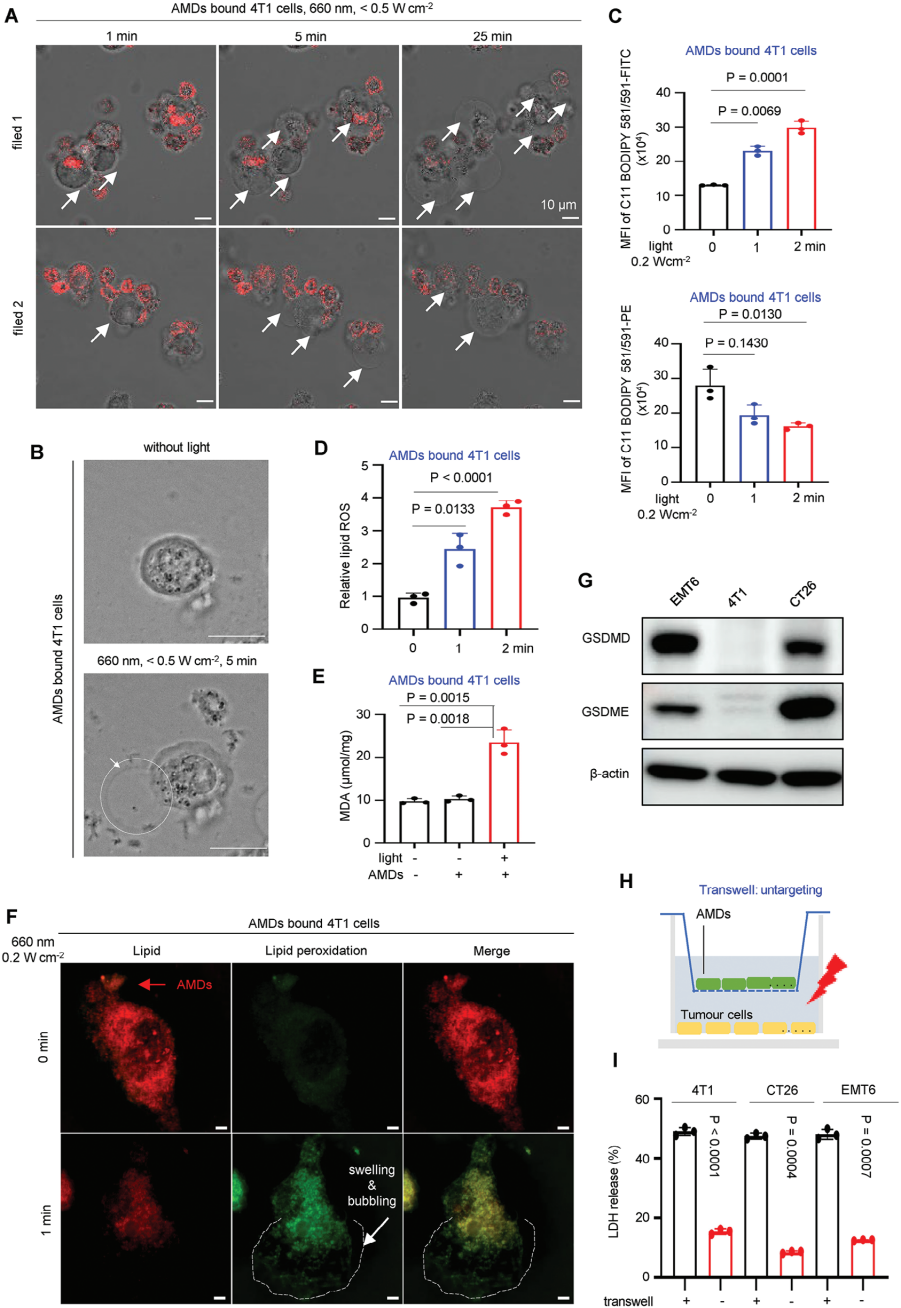
The light‐controlled PMR and pore‐forming activity of AMDs. A) Representative time‐lapse images of AMDs bound 4T1 cells under LSM (laser, 660 nm, c.a., 0.35 W cm^−2^). Scale bars, 10 µm. Red fluorescence of Ce6 indicated AMDs. White arrows showed the bubbling and swelling morphology of 4T1 cells at representative time points. B) Representative images show that AMDs bound to 4T1 cells, causing them to produce bubbles in the presence of light. The bubbles are visible inside the white circles. C) The lipids peroxidation in AMDs bound 4T1 cells with indicated times were analyzed by FACS (660 nm, 0.2 W cm^−2^). The lipid peroxidation was detected by BODIPY 581/591 C11 (10 µm, 30 min incubation. top, FITC channel, oxidized BODIPY. bottom, PE channel, non‐oxidized BODIPY). D) Determining relative lipid ROS by quantitating the fluorescence intensities and calculating the ratio of intensity in FITC channel to the intensity in PE channel. Data (C,D) are shown as mean ± s.d. (*n* = 3, independent experiments, two‐sided Student's *t*‐test). E) The MDA concentration of AMDs bound 4T1 cells treated with or without light (660 nm, 0.2 W cm^−2^, 10 min). Data are shown as mean ± s.d. (*n* = 3, independent experiments, two‐sided Student's *t*‐test). F) Representative confocal images of the lipid peroxidation in AMDs bound 4T1 cells (660 nm, 0.2 W cm^−2^, 0 or 1 min). The red arrow indicates the AMDs. The white arrow indicated the cell swelling and bubbling. The white dash line indicates the bubble. The lipid peroxidation was detected by BODIPY 581/591 C11 (10 µm, 30 min incubation. Red, non‐oxidized BODIPY; Green, oxidized BODIPY). Scale bars, 10 µm. G) Immunoblotting assays of the GSDME and DSDMD expression in indicated tumor cells. H) Schematic representation of the mechanism of the transwell assay by artificially blocking the binding between AMD and tumor cells. In the untargeting mode, AMDs are placed on the transwell and the tumor cells to the bottom of the cell chamber, thus preventing contact between AMDs and cancer cells. I) LDH release of tumor cells (4T1, EMT6, and CT26) treated with AMDs (660 nm, 1.0 W cm^−2^, 10 min) in transwell blocking assay or binding assay. All data are presented as mean ± s.d. (*n* = 3, independent experiments, two‐sided Student's *t*‐test).

All cell membrane disruptors, whether natural or artificial, bind to the plasma membrane prior to performing their membranolytic function.^[^ ^5^, ^12^, ^13^ ^]^ In the design of AMDs, macrophages served as carriers to target and anchor the plasma membrane of cancer cells (Figure 2N), as studies had shown that macrophages and LNPT‐macrophages could bind tumor cells through integrins and VCAM‐1 (Figure 2O).^[^ ^7^, ^8^, ^23^, ^24^ ^]^ High‐resolution mass spectrometry (HRMS) analysis showed that AMDs had inherited most of the integrins and directed antigens after liquid nitrogen treatment (**Figure** **3**A), however, considering that the surface of AMDs undergoes a series of chemical modifications, whether these integrins retained their binding ability was unknown. Further FACS analysis revealed that the AMDs could be labeled by fluorescent antibodies of integrins, indicating that the integrins on the AMDs were still functional in recognition (Figure S3B, Supporting Information). Then, the AMDs were incubated with the VCAM‐1 positive tumor cells. After washing with PBS, nearly all tumor cells were surrounded by AMDs, suggesting AMDs can bind VCAM‐1 positive cells (Figure 2P; Figure S3C, Supporting Information). Moreover, we discovered that AMDs could not bind tumor cells if the VCAM‐1 on the tumor cells was neutralized using the corresponding antibodies, suggesting that recognition of integrins and VCAM‐1 is essential for binding (Figure S3D,E, Supporting Information). For VCAM‐1 negative healthy cells (e.g., TC‐1, AML12, and TCMK‐1),^[^ ^8^ ^]^ the binding by AMDs is also barely observed. Finally, we discovered the existence of “don't eat me” signals on AMDs, which grants them the capability to resist phagocytosis clearance (Figure S3A,B, Supporting Information).^[^ ^19^, ^25^ ^]^

After evaluating the binding capacity of AMD and tumor cells, we began investigating the specific insertion of Ce6 into the plasma membrane. As a lipophilic small molecule, free Ce6 has the ability to insert into any membranous organelle (e.g., mitochondria, endoplasmic reticulum, Golgi apparatus) (Figure S4A–D, Supporting Information). In contrast to free Ce6, the AMDs were found to remain only on the surface of cancer cells. This is due to the micron‐level site‐blocking effect and antigen‐directed binding (Figure 2N–P). Thus, when the AMDs‐bound 4T1 cells were observed under a laser confocal microscope (LSM), the images revealed that the fluorescence of Ce6 was specifically distributed on the plasma membrane at the contact interface between the cells (Figure 2P,Q; Figure S3A, Supporting Information). Notably, although the laser intensity of LSM was very low (c.a. 0.35 Wcm^−2^), with increasing exposure time, all AMDs bound 4T1 cells swelled and bubbled with increasing exposure time. However, in the absence of light, the cells did not bubble (Figure 3A,B; Video S1, Supporting Information), suggesting that the plasma membrane‐specific Ce6 insertion successfully mediated PMR under light (Figure 3A,B). The mechanism of AMD‐mediated PMR was then investigated in gasdermin‐deficient 4T1 cells. The C11‐BODIPY581/591 is a fluorescence ratio probe of lipid peroxidation and shows good spectral separation of the non‐oxidized (595 nm) and oxidized (520 nm) forms.^[^ ^8^ ^]^ We exposed the AMDs bound C11‐BODIPY581/591 labeled 4T1 cells to 0.2 Wcm^−2^ of red light. After irradiation, FACS analysis showed that the intensity of reactive oxygen species (ROS) increased gradually with longer exposure to light, as observed in the FITC channel (Figure 3C). Additionally, after only 2 min of light exposure, the degree of lipid peroxidation was significantly higher, as indicated by the PE channel (Figure 3C). Furthermore, the fluorescence intensity ratio of FITC to PE was used to indicate the accumulation of ROS in lipids, as shown, the light treatments increased the ROS accumulation in lipids (Figure 3D). Thus, the MDA index increased when the AMD‐bound 4T1 cells were treated with light (Figure 3E). Finally, we imaged the light‐driven PMR in AMDs‐bound C11‐BODIPY581/591 labeled 4T1 cells, after light treatment, significant lipid peroxidation was observed along with cell swelling (Figure 3F), confirming that our AMDs‐mediated PMR was caused by lipid peroxidation.

### 2.3 The Binding Between AMD and Tumor Cells Is the Key for Light‐Driven PMR

After confirming that PMR operates through a ROS‐induced lipid peroxidation reaction, we investigated whether light‐driven PMR requires binding between AMD and tumor cells. As introduced, PMR resulted in the release of certain soluble, cytosolic cellular contents. Lactate dehydrogenase (LDH) is a stable enzyme that is present in all cell types and is rapidly released into the cell medium when the plasma membrane is damaged.^[^ ^1^, ^7^, ^8^ ^]^ Therefore, LDH is the most widely used marker in the study of PMR‐related cell death. Here we used a transwell assay to test whether PMR still occurred when the binding was absent (Figure 3H).^[^ ^8^ ^]^ For gasdermin negative 4T1 cells (Figure 3G), LDH release was significantly reduced in transwell treated groups under 660 nm light, indicating that the probability of PMR was extremely low in the absence of binding. Notably, in CT26 and EMT6 cells (Figure 3G), which are highly sensitive to the ROS‐mediated caspase‐GSDM pathway (e.g., caspase‐3‐GSDME or caspase‐1‐GSDMD),^[^ ^5^ ^]^ the low LDH release confirmed that the ROS produced by AMDs, whether via the caspase pathway or the lipid peroxidation pathway, would not trigger PMR without the aid of binding (Figure 3I). All data showed that the binding between AMD and tumor cells is the key for light‐driven PMR.

### 2.4 The AMDs‐Mediated Irreversible and Fast Lytic Cell Death in a Catalytic Manner

**Figure 4:**
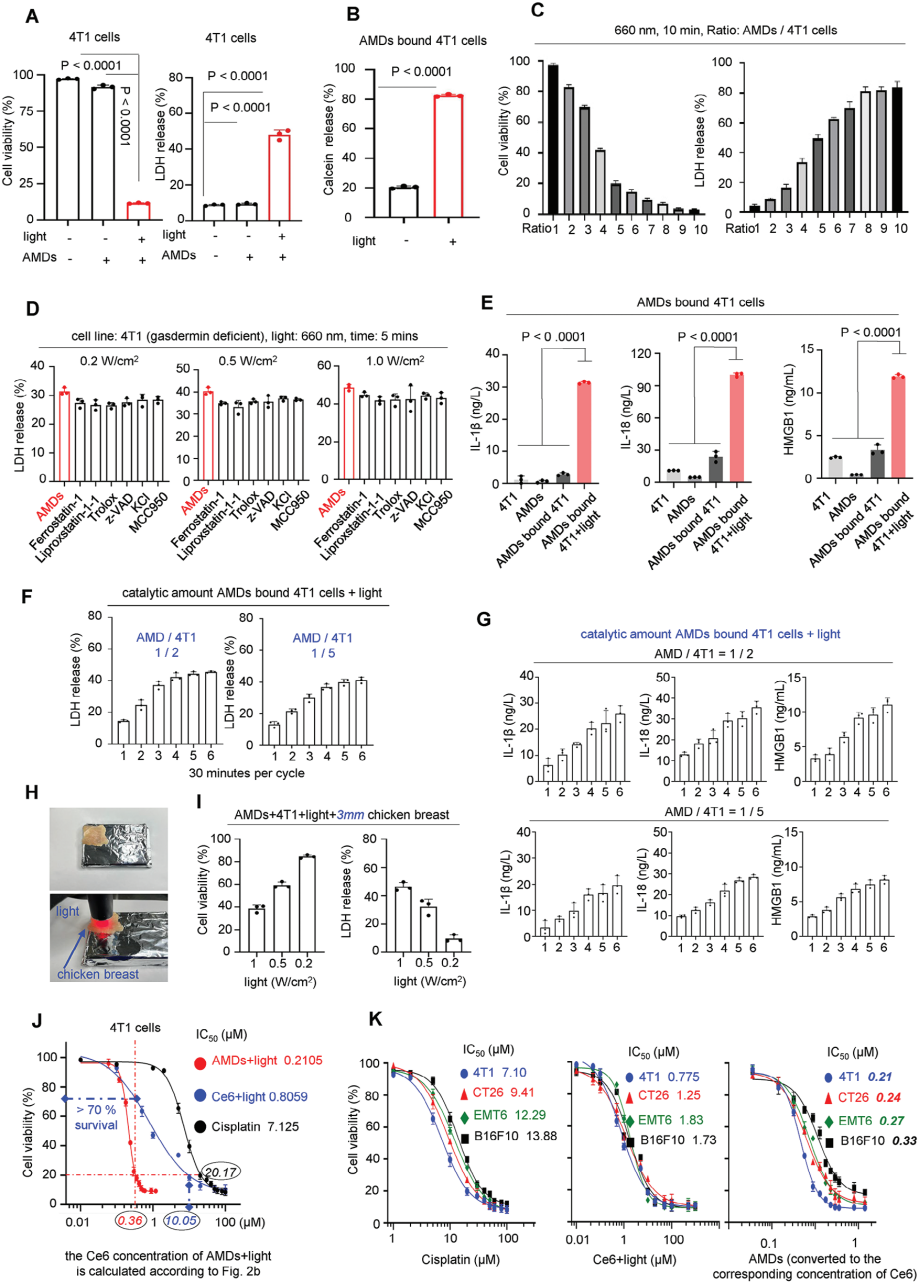
AMDs‐mediated cell death in vitro. A) The CCK‐8 (left) and LDH release assays (right) of AMDs bound 4T1 cells treated with or without light (660 nm, 1.0 W cm^−2^, 10 min). Data are shown as mean ± s.d. (*n* = 3, independent experiments, two‐sided Student's *t*‐test). B) The Calcein release of AMDs bound 4T1 cells treated with or without light (660 nm, 1.0 W cm^−2^, 10 min). Data are shown as mean ± s.d. (*n* = 3, independent experiments, two‐sided Student's *t*‐test). C) The CCK‐8 (left) and LDH release assays (right) of AMDs mediated cell death in 4T1 cells treated with light (660 nm, 1.0 W cm^−2^, 10 min) under a series ratios of AMDs to 4T1 cells. Data are shown as mean ± s.d. (*n* = 3, independent experiments). D) LDH release of AMDs bound 4T1 cells (660 nm, 0.2, 0.5, or 1.0 W cm^−2^, 5 min) pre‐treated with antioxidants (e.g., Ferrostatin‐1, Liproxstatin‐1, Trolox), pan‐caspase inhibitor z‐VAD, KCl, and NLRP3 inhibitor MCC950. All data are presented as mean ± s.d. (*n* = 3, independent experiments). E) Secretion of IL‐1β, IL‐18, HMGB‐1 from 4T1, AMDs, AMDs bound 4T1, or AMDs bound 4T1 treated with light (660 nm, 1.0 W cm^−2^, 10 min) were tested by corresponding ELISA kits. Data are shown as mean ± s.d. (*n* = 3, independent experiments, two‐sided Student's *t*‐test). F) The catalytic killing of AMDs, 660 nm, 1.0 W cm^−2^, 30 min per cycle. LDH release was tested after indicated irradiation times. The ratio of AMDs to 4T1 cells was 1/2 or 1/5. All data are presented as mean ± s.d. (*n* = 3, independent experiments, two‐sided Student's *t*‐test). G) The cytokines release in the catalytic killing assays. H) Schematic representation of the use of chicken breast to simulate AMDs responding to red light‐mediated cell death under deep tissue. I) The CCK‐8 and LDH release assays of AMDs bound 4T1 cells treated with indicated light (660 nm, 10 min) in the presence of 3 mm breast chicken tissue. Data are shown as mean ± s.d. (*n* = 3, independent experiments). J) The cytotoxicity of AMDs+light‐treated, Ce6 alone+light‐treated, and Cisplatin treated 4T1 cells. K) The cytotoxicity of AMDs+light‐treated, Ce6 alone+light‐treated, and Cisplatin treated tumor cells. Data (J,K) are shown as mean ± s.d. (*n* = 3, independent experiments).

The AMDs successfully mediated PMR in pyroptosis‐deficient cells under light. However, it is still unclear whether these damages can lead to efficient cell death. The efficiency of AMDs‐mediated lytic cell death was tested using the CCK8 and LDH assays. After light treatment, cell viability significantly decreased and LDH increased in 4T1 cells bound to AMDs, indicating the induction of lytic cell death by AMDs+light treatment (**Figure** **4**A). Furthermore, it was found that AMDs alone did not exhibit cytotoxicity; The lytic cell death mediated by AMDs was dependent on both light and AMDs (Figure 4A). To exclude the almost non‐existent influence of the AMDs themselves on LDH release, the Calcein_AM labeled 4T1 cells were prepared to repeat the above tests.^[^ ^3^ ^]^ After irradiation, a significant release of Calcein was observed, confirming that the AMDs mediated PMR resulted in lytic cell death (Figure 4B). Notably, the intensity of lytic cell death described above was positively correlated with the addition of AMDs, and as the ratio of AMDs to 4T1 cells increased, there was an increase in lytic cell death under light (Figure 4C). As previously mentioned, binding is essential for AMD‐mediated PMR. Therefore, it is difficult to observe AMD‐mediated light‐driven PMR and cell death in healthy cells due to the lack of sufficient binding (Figure S5A, Supporting Information).

Additionally, we discovered that light‐induced lytic cell death caused by AMDs is rapid and irreversible (Figure 4D). Although we demonstrated that AMDs‐mediated PMR resulted from lipid peroxidation, the addition of antioxidants did not prevent LDH release under different light intensities. This suggests that once the plasma membrane‐specific Ce6 is activated by light, the ROS generated in the binding region become saturated or exceed the substrates to be oxidized and the lytic cell death mediated by AMDs was irreversible (Figure 4D). Although studies have demonstrated that ROS can induce necrosis, a classical cell death characterized by PMR, by activating NLRP3 or caspase‐related pathways,^[^ ^26^ ^]^ both the NLRP3 inhibitor MCC950 and the pan‐caspase inhibitor z‐VAD showed little effect on LDH release (Figure 4D). This indicates that the PMR mediated by ADMs was so rapid that lytic cell death was completed before NLRP3 and caspases were activated. The immunoblotting assays also demonstrated that neither NLRP3 nor caspase‐1 was activated during AMDs‐mediated lytic cell death (Figure S5B, Supporting Information). Notably, the aforementioned rapid and irreversible lytic cell death resulted in the secretion of numerous pro‐inflammatory cytokines, particularly HMGB‐1, indicating that AMDs‐mediated cell death under light was immunogenic (Figure 4E).

Better than other membranolytic molecules,^[^ ^12^, ^13^ ^]^ our AMDs exhibited catalytic killing ability. The 4T1 cells were incubated with a catalytic amount of AMDs at a ratio of 1/2 or 1/5. Subsequently, pulsed light treatments were applied, resulting in continuous cell death. The levels of LDH, IL‐1β, IL‐18, and HMGB‐1 increased with the light inputs, indicating that the catalytic amount of AMDs moved to other 4T1 cells to perform the next cell lysis when PMR had formed in a target area (Figure 4F,G). Furthermore, the catalytic amount of AMDs bound to 4T1 cells were treated with pulsed light, then, the samples were subjected to a CCK8 assay at 6 and 12 h after light treatment. The results showed no significant difference in cell death between the two assay conditions, indicating the sustained release of LDH and inflammatory factors observed in the above experiments is not caused by cell death at different time points (Figure S6A, Supporting Information). The catalytic killing ability gave AMDs excellent performance in deep tissues, even when light was blocked by 3–12 mm of chicken flesh, AMDs‐mediated cell lysis could be detected (Figure 4H,I; Figure S6B,C, Supporting Information). Furthermore, the high potency of AMDs resulted in a significantly higher killing capacity than Ce6 alone and the classical chemotherapeutic drug cisplatin. The concentrations of AMDs, Ce6‐alone, and cisplatin in mediating 80% 4T1 cell death were 0.36, 10.05, and 20.17 µm, respectively (Figure 4J). For other tumor cells, AMDs+light treatment also exhibited greater cytotoxicity, with IC50 values typically one‐fifth and one‐fiftieth of those of Ce6 and cisplatin alone, suggesting that AMDs+light treatment has potential in the treatment of drug‐resistant tumors (Figure 4K).

### 2.5 Tumor Targeting of the AMDs in Mice and Light‐Controlled Tumor Specific PMR

**Figure 5:**
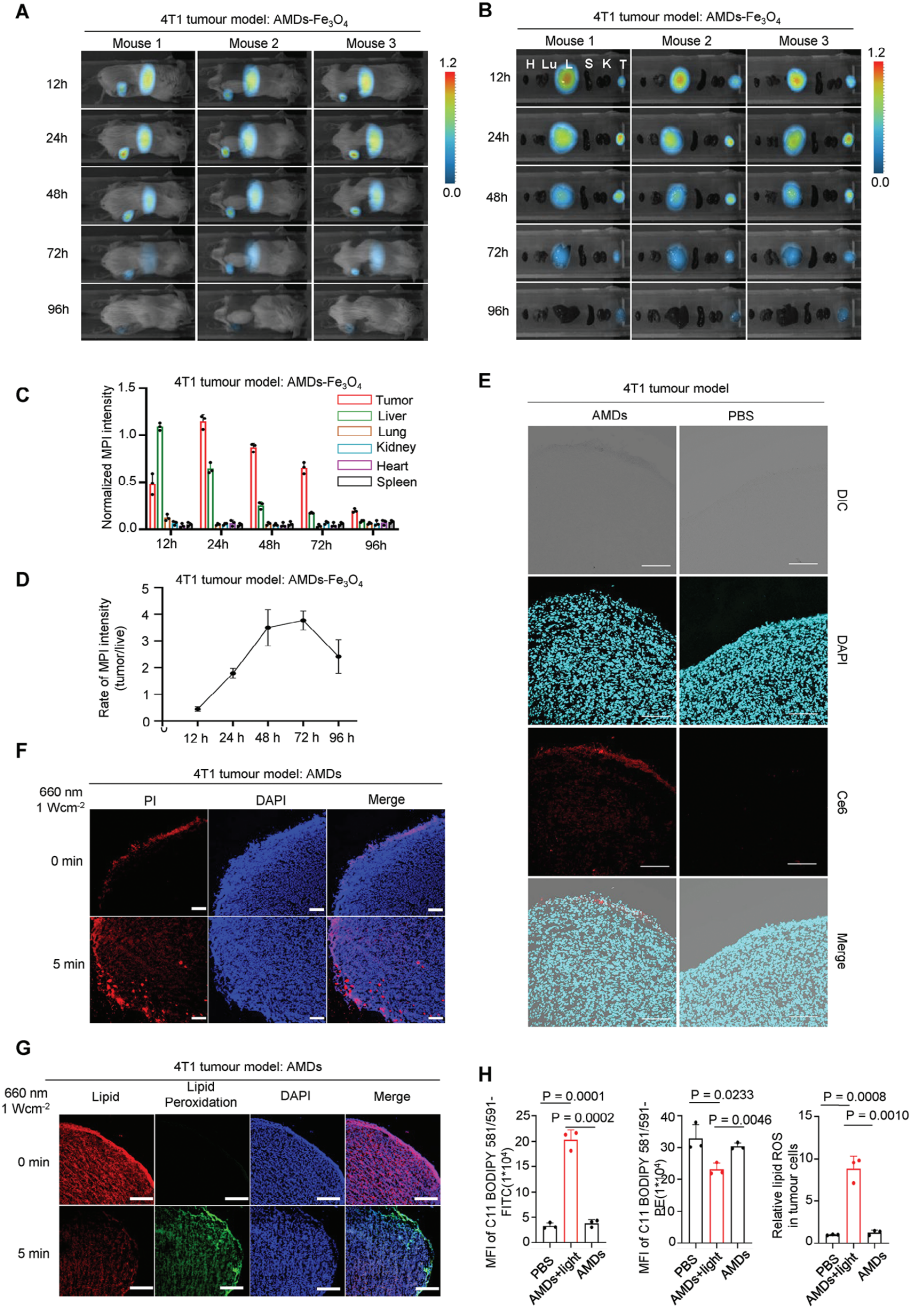
Tumor targeting of AMDs and light‐controlled specific PMR in vivo. A) The MPI images of three independent 4T1 tumor bearing mice intravenously injected with AMDs ‐Fe_3_O_4_ for 96 h. B) The biodistribution of AMDs ‐Fe_3_O_4_ at different time points. H, heart, Lu, lung, S, spleen, L, liver, K, kidney, T, tumor. C) Average intensity of AMDs‐Fe_3_O_4_ in the corresponding organs of the indicated tumor models at a series of time points. D) The tumor‐to‐liver ratio of MPI intensity at different time points. Data (C,D) are shown as mean ± s.d. (*n* = 3 mice). E) Representative confocal images of tumor sections showed the penetration of AMDs (5 x 10^6^ units per mouse) in 4T1 tumor‐bearing mice 24 h after intravenous injection. Scale bar, 100 µm. F) Representative tumor section fluorescence images of propidium iodide‐positive cells of 4T1 tumor‐bearing mice treated with AMDs+light (660 nm, 1.0 W cm^−2^, 5 min). Scale bars, 100 µm. Propidium iodide (2.5 mg kg^−1^) intravenously injected into the mice at 24 h after the last round of light treatment. G) Representative tumor section fluorescence images of lipid peroxidation based on BODIPY® 581/591 C11 stained 4T1 tumor‐bearing mice treated with AMDs+light (660 nm, 1.0 W cm^−2^, 5 min). Scale bars, 100 µm. H) Tumor lipid peroxidation of 4T1 tumor‐bearing mice treated with PBS, AMDs with or without light (660 nm, 0.5 W cm^−2^, 5 min), *n* = 3 for indicated groups. Mean fluorescence intensities (MFI) of oxidized BODIPY (FITC channel) or, non‐oxidized BODIPY (PE channel) and the relative lipid ROS in 4T1 tumors with indicated treatments. The relative lipid ROS was calculated as the ratio of oxidized and non‐oxidized BODIPY MFI.

As previously stated, our AMDs were obtained from mannose‐Ce6 engineered immune cells. Although immune cells, particularly macrophages, and LNPT‐macrophages, can target tumors through antigen‐directed navigation,^[^ ^7^, ^8^, ^27^, ^28^ ^]^ it is uncertain whether our Ce6 engineering approach eliminated their targeting. Therefore, we aimed to investigate the in vivo biodistribution of AMDs. To preserve the original state of the antibodies on the surface of the AMDs, superparamagnetic iron tetraoxide nanoparticles (Fe_3_O_4_) were chosen to label AMDs for magnetic particle imaging (MPI).^[^ ^7^, ^8^ ^]^ The 4T1 tumor‐bearing mice were employed and the Fe_3_O_4_‐labelled AMDs (AMDs‐Fe_3_O_4_) were injected intravenously. The biodistribution of the AMDs could then be observed by MPI at different time points and subsequent statistical analysis. To our delight, AMDs demonstrated tumor‐targeting for up to 96 h in 4T1 tumor‐bearing mice (**Figure** **5**A; Figure S7A, Supporting Information). Specifically, combined images and magnetic signal statistical analyses showed that significant tumor enrichment was observed 12 h after AMDs entered the circulatory system of the mice, however, there were also more AMDs in the liver at this time (Figure 5B,C). Subsequently, AMDs in the liver decreased rapidly, whereas AMDs in the tumors peaked at 24 h post‐injection and then gradually decreased (Figure 5B,C). Additionally, the hepatic tumor ratio of AMDs started to increase rapidly 12 h after injection, followed by a quick decrease after reaching its peak (c.a. 4.0) at h 72 (Figure 5D). We sacrificed the AMD‐treated mice at the 24‐h point, harvested the tumors, and analyzed the penetration. As shown, using the fluorescence of Ce6, we found that AMDs could infiltrate into the tumors (Figure 5E). Although the above data showed that our AMDs were only tumor‐selective and not specific, the light‐driven PMR was tumor‐specific as the light was focused on the tumor. We then used 660 nm light on AMDs treated 4T1 tumor‐bearing mice to analyze PMR and lipid oxidation in vivo. Propidium iodide (PI) is a nuclear stain that cannot pass through living cell membranes, but it can pass through damaged cell membranes to stain the nucleus.^[^ ^1^, ^7^, ^8^ ^]^ After light treatment, we administered PI intravenously to the AMD‐treated tumor‐bearing mice and analyzed the tumors by fluorescence co‐location sectioning, the results showed a clear increase signal of PI in light‐treated mice, indicating the PMR happened (Figure 5F). We also took tumor sections and performed lipid peroxidation probe staining. The images showed significant lipid oxidation, indicating that the PMR was caused by lipid peroxidation in vivo (Figure 5G). Furthermore, we prepared the single cell suspension of AMDs(i.v.)+light treated tumors, the corresponding FACS analysis confirmed the lipid peroxidation and ROS accumulation in tumors (Figure 5H; Figure S7B, Supporting Information).

### 2.6 The AMDs Boost Antitumor Immune Response in Pyroptosis‐Deficient Tumor Bearing Mice

**Figure 6:**
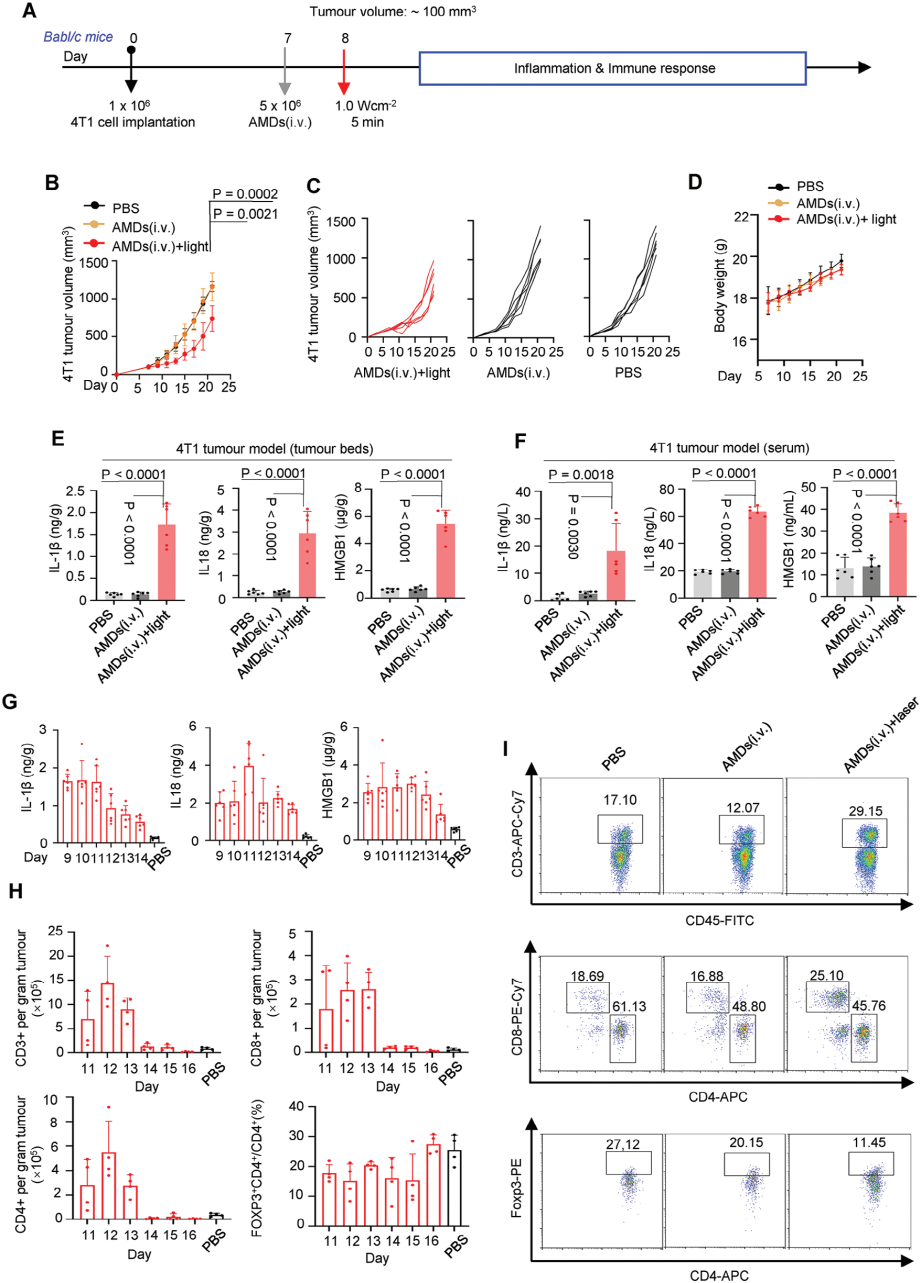
The AMDs‐mediated PMR under light induces antitumor immune response. (A–F) Analysis of the antitumor effects in 4T1 tumor‐bearing mice treated with AMDs+light. A) AMDs+light treatment scheme in BALB/c mice implanted subcutaneously with 4T1 cells (1 x 10^6^ cells per mouse). The tumor‐bearing mice were intravenously injected with AMDs (i.v., 5 x 10^6^ units per mouse) on day 7 and then treated with light (660 nm, 1.0 W cm^−2^, 5 min) on days 8. B) Average tumor volume. C) Tumor volume of an individual mouse. D) The body weight. E,F) The concentrations of IL‐18, IL‐1β and HMGB1 concentrations in the tumor homogenates (E) and serum (F) of 4T1 tumor‐bearing mice treated with AMDs+light at day 9 (*n* = 6 mice, as shown in the figures for each group). Data are shown as mean ± s.d. (*n* = 6 mice, two‐sided Student's *t*‐test). G) Monitoring the cytokines of tumor beds by ELISA for 6 days H) Quantification of tumor‐infiltrating lymphocytes including CD3^+^, CD4^+^, CD8^+^ T cells, and FOXP3^+^CD4^+^ regulatory T cells from mice treated with AMDs+light was monitored continuously for 6 days (*n* = 4 mice per group, as shown in the figures for each group). I) Representative FACS analyses of tumor‐infiltrating lymphocytes in indicated groups.

Since light‐driven PMR was tumor‐specific, we investigated the anti‐tumor effects of AMDs in gasdermin‐deficient tumor‐bearing mice. 4T1 tumor‐bearing mice (BALB/c mice) were intravenously injected with the AMDs (5 × 10^6^ units per mouse, c.a., 75 µg Ce6 per mouse) on day 7, followed by a single light exposure for 5 min (**Figure** **6**A). In PBS and AMD(i.v.) alone treated mice, all tumor volumes increased rapidly to 20‐fold higher than the initial value within 2 weeks (Figure 6B,C). In addition, in Ce6(i.t.)+light treated mice, although triple Ce6 (200 µg per mouse) was injected intratumorally, little significant anti‐tumor effect was observed (Figure S8A–C, Supporting Information). Notably, in AMDs(i.v.)+light treated mice, tumor growth was delayed within 2 weeks after the end of treatment, but then rapidly rebounded (Figure 6B,C).

Recent studies have shown that antitumor systems inducing immunogenic cell death may not have high initial therapeutic efficacy.^[^ ^1^, ^2^, ^3^, ^7^, ^8^ ^]^ However, the inflammation of immunogenic cell death can be used to enhance T‐cell infiltration, thereby sensitizing antitumor immunotherapy.^[^ ^1^, ^2^, ^3^, ^7^, ^8^ ^]^ Since the AMDs‐mediated cell death exhibits characteristics of immunogenic cell death, therefore, an inflammatory and immune analysis was performed. In the tumor beds of AMDs(i.v.)+light‐treated mice, IL‐1β, IL‐18, and HMGB‐1 were significantly increased, while the cytokines in PBS‐treated and AMDs(i.v.)‐alone treated mice were low, indicating that the tumor‐specific PMR induced local inflammation and the inflammation‐inducing ability was both AMDs and light‐dependent (Figure 6E). Moreover, when serum cytokines were tested, they decreased almost 100‐fold compared to the concentration in the tumor beds, indicating that the inflammation‐inducing ability was tumor‐specific (Figure 6F). As the inflammation induced by AMDs(i.v.)+light was mainly concentrated in the tumor beds and not systematically, the body weights of all AMDs(i.v.)+light treated mice gradually increased (Figure 6D). After light treatment, PBS, AMDs(i.v.)+light, and AMDs(i.v.)‐treated mice were sacrificed and lungs, livers, and kidneys were analyzed. Although the biodistribution of AMDs suggests that they are also distributed in healthy tissues (e.g., livers, lungs), it is important to note that red light only irradiates tumors, thus, no damage was found in healthy tissues of AMDs(i.v.)+light treated mice (Figure S9, Supporting Information). The apparent tumor‐specific inflammation lasted for at least 5 days (Figure 6G), successfully recruiting large numbers of T cells to infiltrate the immunodeficient 4T1 tumors. A drastically larger tumor CD3^+^ T cell population was observed in AMDs(i.v.)+light‐treated mice than in tumors from PBS‐treated mice and AMDs(i.v.)‐alone treated mice (Figure 6H; Figure S8D,E, Supporting Information). Among the T cells, both the CD4^+^ and CD8^+^ subpopulations were ≈10‐fold larger (Figure 6I; Figure S8E, Supporting Information). The immune response of CD3^+^, CD4^+^, and CD8^+^ T cells peaked at day 12 and disappeared from day 14 (Figure 6H). The percentage of CD4^+^Foxp3^+^ T regulatory (Treg) cells, which are negative regulators of antitumor immunity, decreased in the tumors (Figure 6H; Figure S8E, Supporting Information). Dendritic cells (DCs) are initiators of adaptive immune responses,^[^ ^7^, ^8^, ^29^ ^]^ in AMDs(i.v.)+light‐treated mice, the CD80 and CD86 positive DCs are significantly increased in lymph nodes, indicating the PMR in tumors activated adaptive immune responses (Figure S8F–H, Supporting Information).

**Figure 7:**
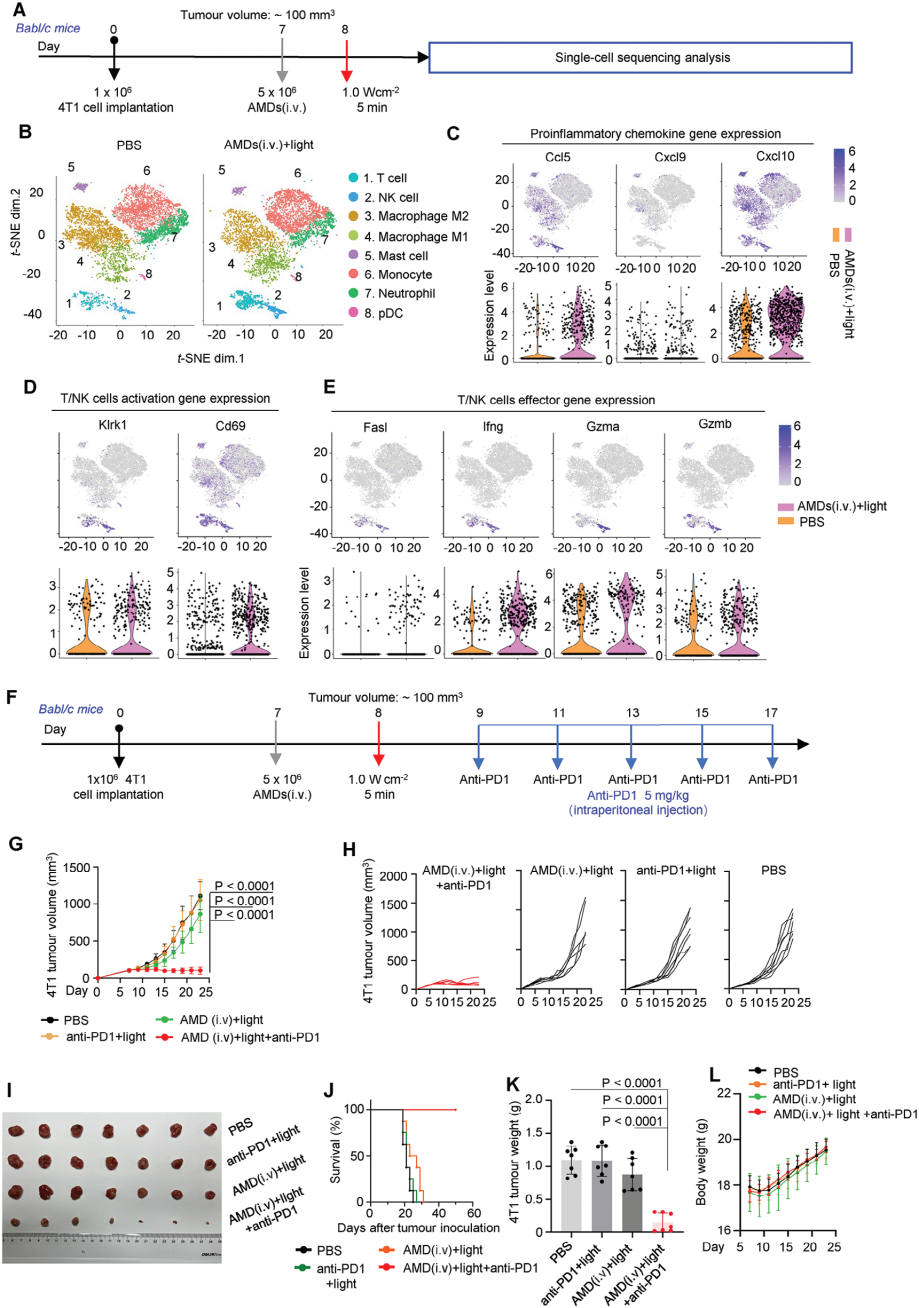
The single‐cell sequencing analysis and combination therapy. (A–E) The single‐cell sequencing analysis of PBS and AMDs+light treated mice. A) The treatment scheme. B) The *t*‐distributed stochastic neighbor embedding (t‐SNE) density plots of CD45^+^ cells randomly sampled from each group are shown. NK, natural killer. C) Proinflammatory chemokine gene expression. D) T/NK cells activation gene expression. E) T/NK cells effector gene expression. (F–L) Antitumor immunotherapy of AMDs+light synergized with anti‐PD‐1 antibody. F) AMDs+light synergizes with the anti‐PD‐1 treatment scheme in BALB/c mice implanted subcutaneously with 4T1 cells (1 x 10^6^ cells per mouse). The tumor‐bearing mice were intravenously injected with AMDs (i.v., 5 x 10^6^ units per mouse) on day 7 and then treated with light (660 nm, 1.0 W cm^−2^, 5 min) on day 8, followed by anti‐PD‐1 therapy. G) Average tumor volume. Data are shown as mean ± s.d. (*n* = 7, independent experiments, two‐sided Student's *t*‐test). H) Tumor volume of an individual mouse. I) Photographs of representative tumors on day 23. J) Survival curves of 4T1 tumor‐bearing mice (8 mice per group, log‐rank test; *p* < 0.0001). K) Average tumor weight on day 23. L) The body weight. Data are shown as mean ± s.d. (*n* = 7, independent experiments, two‐sided Student's *t*‐test).

Although the above FACS analysis showed a significant improvement in T cell infiltration, the function of these T cells was still unknown. Therefore, single‐cell RNA sequencing on CD45^+^ leukocytes was performed to further investigate the inflammation and immune response in AMDs(i.v.)+light treated 4T1 tumor‐bearing mice (**Figure** **7**A). In terms of inflammation, the population of monocytes and M1 macrophages increased after AMDs(i.v.)+light treatment (Figure 7B). In addition, the gene expression of pro‐inflammatory cytokines (e.g., Ccl5, Cxcl9, and Cxcl10) increased in AMDs(i.v.)+light treated mice compared to PBS treated mice (Figure 7C).^[^ ^30^ ^]^ All these phenomena suggested that AMDs‐mediated tumor‐specific PMR and lytic cell death were pro‐inflammatory. In terms of immune responses, the infiltration of T cells and NK cells was improved after AMDs(i.v.)+light treatment (Figure 7B). More importantly, genes important for lymphocyte activation (Cd69 and Klrk1) were upregulated in tumors treated with AMDs(i.v.)+light (Figure 7D).^[^ ^31^ ^]^ Anti‐tumor effector genes (Ifng, Gzma, Gzmb, and Fasl) were also upregulated in tumors treated with AMDs(i.v.)+light (Figure 7E),^[^ ^32^ ^]^ whereas the protumoral or immunosuppressive genes Csf1, Vegfa, Arg1, Cd274 (encoding PD‐L1), and Pdcd1lg2 (encoding PD‐L2) were downregulated to varying degrees (Figure S10, Supporting Information).^[^ ^30^, ^31^, ^32^, ^33^ ^]^ These immunological changes confirmed the potent antitumor effects of AMDs(i.v.)+light treatment.

After confirming that AMDs could induce a strong T‐cell‐based immune response in gasdermin‐deficient tumors. We investigated whether anti‐PD‐1 therapy could synergize the above immune response to eradicate tumors.^[^ ^33^, ^34^ ^]^ After five minutes of light exposure, anti‐PD‐1 antibodies were administered five times by intraperitoneal injection (Figure 7F). To our satisfaction, tumor shrinkage occurred in the AMDs(i.v.)+light+PD‐1 treated mice, however, as the broad type 4T1 tumors were unresponsive to anti‐PD‐1 therapy, no tumor shrinkage was found in the PD‐1 alone treated mice (Figure 7G–L). No weight loss was observed in all treated mice and the survival benefit was evident in AMDs(i.v.)+light+PD‐1 treated mice (Figure 7J–I). Thus, our AMDs enhanced anti‐tumor immune T‐cell infiltration and enhanced anti‐PD‐1 therapy to eradicate the pyroptosis‐deficient 4T1 tumors.

## 3 Discussion

This study shows that our AMDs effectively mediated PMR and enhanced the T‐cell‐based immune response, thereby improving the response to anti‐PD‐1 therapy. However, the direct anti‐tumor effects of AMDs‐mediated programmed cell death (PMR) appear to be weak. This reflects the difference between the inflammatory and immunological features caused by artificial PMR and natural PMR‐mediated cell death. Fortunately, the AMD has been designed and constructed in a modular fashion, which offers the potential for future iteration and modification of its function. Additionally, the study demonstrates that a bioinspired membrane disruptor efficiently mediates PMR and antitumor immune function in pyroptosis‐deficient tumors in a catalytic and controllable manner. These findings provide new insights into the design of artificial membrane disruptors to overcome the natural limitations of manipulating cell lysis. An artificial membrane disruptor was used to investigate the distinctive immunogenicity of cell death characterized by PMR, independent of intrinsic mechanisms. Our in vivo application of AMDs confirmed the tumor‐specific PMR's ability to induce antitumor immune function. Although the direct antitumor effect of AMDs was unsatisfactory in our study, the PMR‐induced T‐cell‐based immune response could synergize with anti‐PD‐1 therapy to cure cancer. Thus, a drug that can induce PMR may enhance the effectiveness of cancer immunotherapy in clinical settings.

## 4 Experimental Section

### Data Reporting

No statistical methods were used to predetermine sample size. The experiments were not randomized, and investigators were not blinded to allocation during experiments and outcome assessment. Ethical compliance with the IACUC protocol was maintained. In none of the experiments did the size of the tumor graft surpass 2 cm in any two dimensions, and no mouse had severe abdominal distension (≥10% increase in original body weight), as outlined by the Animal Experimental Ethical Inspection committee of the Laboratory Animal Centre, Wenzhou Medical University (ID number: xmsq2021‐0530).

### Materials

Ferrostatin‐1 and Liproxstatin‐1 were purchased from Selleck. Ac4ManNAz, Calcein‐AM, and Trolox were purchased from Sigma–Aldrich. Z‐VAD‐FMK and MCC950 were purchased from MedChemExpress. ProLong Diamond Antifade Mountant with DAPI, PageRuler Prestained Protein Ladder (26 617), Image‐iT Lipid Peroxidation Kit, and Singlet Oxygen Sensor Green reagent was obtained from Thermo Scientific. DiIC18(3) was obtained from Yeasen Biotechnology. Propidium iodide and Lipid Peroxidation MDA Assay Kit were purchased from Beyotime.

Anti‐GSDME (ab215191) and anti‐GSDMD (ab209845) were from Abcam. Anti‐rabbit IgG (H+L), F(ab')2 Fragment (Alexa Fluor 488 Conjugate) (CST4412), and Anti‐β‐Actin (CST8457) were purchased from Cell Signaling Technology.

Cell viability and LDH release assays were determined by using the Cell Counting Kit‐8 (CCK‐8, DOJINDO) and Cytotoxicity LDH Assay Kit (DOJINDO), respectively. For fluorescence‐activated cell sorting (FACS) analyses of tumor‐infiltrating lymphocytes, FITC anti‐mouse CD45 antibody‐S18009F, APC/Cyanine7 anti‐mouse CD3‐17A2, APC anti‐mouse CD4‐RM4‐4, PE/Cyanine7 anti‐mouse CD8a‐53‐6.7 were purchased from BioLegend. PE FOXP3 monoclonal antibody (FJK‐16s) was obtained from Invitrogen. The InVivoMAb anti‐mouse PD‐1 antibody (clone J43) used for treating 4T1 tumors were obtained from BioXcell.

Enzyme‐linked immunosorbent assay (ELISA) kits for IL‐1β, IL‐6, IL‐10, IL‐18, TNF‐α, TGF‐β, and HMGB1 were purchased from MEIMIAN Biology.

### Cell Lines and Cell Culture Conditions

4T1, CT26, and Raw 264.7 cells were kindly provided by Stem Cell Bank, Chinese Academy of Sciences. EMT6 cells were obtained from the American Type Culture Collection (ATCC). Raw 264.7 cells were grown in Dulbecco's modified Eagle's medium (DMEM, Gibco) supplemented with 10% (v/v) fetal bovine serum (FBS, Gibco) and 1% Pen Strep (Gibco). 4T1, CT26, and EMT6 cells were grown in RPMI 1640 medium (Gibco) containing 10% FBS and 1% Pen Strep. AML12 cells were grown in 1:1 mixture of Dulbecco's modified Eagle's medium and Ham's F12 medium with 0.005 mg mL^−1^ insulin, 0.005 mg mL^−1^ transferrin, 5 ng mL^−1^ selenium, and 40 ng mL^−1^ dexamethasone, 90%; fetal bovine serum, 10%. TCMK‐1 cells were grown in the base medium for this cell line is ATCC‐formulated Eagle's Minimum Essential Medium, Catalog No. 30–2003. To make the complete growth medium, the following components were added to the base medium: fetal bovine serum to a final concentration of 10%. All cells were grown at 37 °C with 5% CO_2_. All cell lines were tested to be mycoplasma‐negative by the standard PCR method. The identity of the cells was frequently checked by their morphological features but had not been authenticated by short tandem repeat (STR) profiling.

### Preparation and Characterization of AMDs—Preparation of AMDs

Ac4ManNAz (125 µm) were incubated with Raw 264.7 (≈10^7^ cells per dish) for 24, 48, and 72 h, followed by gentle washing with PBS or complete medium to produce Raw 264.7‐Ac4ManNAz. Subsequently, Raw 264.7‐Ac4ManNAz (≈10^7^ cells) were resuspended in PBS containing Alkynyl‐Ce6(0.1 µm), ascorbic acid (0.2 µm), and CuSO_4_ (0.2 µm) and incubated for 1 h at 37 °C. Then the same was centrifuged (1000 rpm, 3 min) and washed three times with PBS to obtain precursor of AMDs. Precursor of AMDs (≈10^7^ cells per tube, 1 mL) was immersed in liquid nitrogen, 24 h later, the frozen tubes were thawed at 37 °C, centrifuged at 1000 rpm for 3 min, washed, and resuspended by PBS to generate the AMDs (5 × 10^6^ units per tube, 1 mL).

### Preparation and Characterization of AMDs—Characterization of AMDs

The amount of Ce6 on precursor of AMDs or AMDs (≈10^7^ cells) was evaluated by microplate reader assay. The size distributions and zeta potential of precursor of AMDs or AMDs were characterized by dynamic light scattering (DLS, Malvern Zetasizer Nano S90). For confocal images and flow cytometry, FITC‐DBCO (2 µL, 1 mm) labeled Ac4ManNAz_Raw 264.7 was treated with DiIC18(3) (5 µL, 1 mm) for 0.5 h, followed by washing twice with phosphate‐buffered saline.

### Preparation and Characterization of AMDs—Stability of AMDs under Physiological Conditions

To examine the stability of AMDs under physiological conditions, AMDs (≈10^7^ units) were resuspended in 1 mL PBS at 37 °C, cell counter was used at specified times to assess the change of AMDs in the number.

### Singlet Oxygen Generation

The PBS containing AMDs was added to the 48‐well plate (1 × 10^5^ per well), then Singlet Oxygen Sensor Green(SOSG) reagent (2 µl, 5 mm) was added and incubated for 5 min, followed with light (660 nm, 1.0 W cm^−2^) for different times (1, 5, 10, 20, 30, 60, 120, 240, 480, 600, 900, 1200, 1800, and 2400 s, or 0.5, 1, 2, 3, 4, 5, 6, 7, 8, 9, and 10 min). SOSG was quantified by detecting the fluorescence of singlet oxygen (excitation/emission maxima ≈504/525 nm) with multimode reader (Spark, TECAN). The MFI of SOSG was processed with GraphPad Prism 8.0 (GraphPad Software, CA) to calculate time for half consumption.

### In Vitro Tumor Cell Recognition—Evaluation of the AMDs Bound Tumor Cells

Tumor cells (4T1, CT26 and EMT6, 10^6^ cells per dish) were seeded in glass‐bottom dishes for 6 h and prelabeled with Calcein‐AM (10 µm, 30 min). The above tumor cells were washed twice with PBS to generate the Calcein‐AM labeled 4T1, EMT6, or CT26 cells. The Dil‐labeled AMDs (10^7^ units per dish) were added and co‐incubating for 6 h, unbound AMDs were removed by washing with PBS. Representative images of AMDs bound tumor cells were observed and captured with an Olympus confocal microscope (Olympus FV3000). The image data shown were representative of at least three randomly selected fields.

### In Vitro Tumor Cell Recognition—Evaluation of the AMDs Bound Cells (Tumor Cells or Healthy Cells) by Integrins and VCAM‐1

Tumor cells were seeded in glass‐bottom dishes (4T1, CT26, and EMT6, 10^6^ cells per dish) with a Calcein‐AM (10 µL, 1 mg mL^−1^) containing cell culture medium for 6 h. The above tumor cells were washed twice with PBS to generate the Calcein‐AM labeled 4T1, EMT6, or CT26 cells. The Dil‐labeled AMDs (10^7^ units per dish) was added and co‐incubating for 6 h, unbound AMDs were removed by washing with PBS. Representative images of AMDs bound tumor cells were observed and captured with an Olympus confocal microscope (Olympus FV3000). The image data shown were representative of at least three randomly selected fields. For the detection of AMDs recognition of anti‐VCAM1 treated tumor cells, tumor cells (1 ×1 0^6^ cells per dish) were first seeded in glass‐bottom dishes, 12 h later incubated with cell staining buffer containing anti‐VCAM1(2.5 µg) for 1 h, then washed with PBS three times, and then tested according to the above similar method. The detection of AMDs recognition of TC‐1 cells, AML12 cells, and TCMK‐1 cells was similar to the above method.

### The PMR Activity of AMDs—Microscopic Imaging of Cell Death

To observe the cells morphology with different treatments, 4T1 tumor cells (1 × 10^6^ cells per dish) were seeded in glass‐bottom dishes for 6 h and subjected to AMDs for 6 h (5 × 10^6^ units per dish) and washed twice by PBS, followed with light (660 nm, 1.0 W cm^−2^, 5 min). Representative bright‐field images of cell death were observed and captured with an Olympus confocal microscope (Olympus FV3000). The image data shown were representative of at least three randomly selected fields.

To perform Lipid Peroxidation staining, AMDs bound 4T1 cells in glass‐bottom dishes treated with light (660 nm, 0.2 W cm^−2^, 1 min), then added 10 µm BODIPY 581/591 C11 and incubated for 30 min at 37 °C. Representative confocal images of lipid peroxidation were observed and captured with an Olympus confocal microscope (Olympus FV3000). The image data shown were representative of at least three randomly selected fields. Furthermore, the lipids peroxidation was analyzed by FACS (660 nm, 0.2 W cm^−2^, 1 or 2 min), AMDs bound 4T1 cells treated with light (660 nm, 0.2 W cm^−2^, 1 or 2 min) were resuspended in 1 mL RPMI 1640 complete medium containing 10 µm BODIPY 581/591 C11 and incubated for 30 min at 37 °C. Cells were washed and resuspended in 500 µL fresh PBS (Gibco), then analyzed by flow cytometry using Agilent Novocyte.

### The PMR Activity of AMDs—Lipid Peroxidation Assessed by MDA Assay

AMDs bound 4T1 cells in 6‐well plate treated with light (660 nm, 0.2 W cm^−2^, 1 min). Cell lysis was performed by adding 100 µL lysis buffer per million cells. After lysis, 100 µL of supernatant was obtained by centrifugation at 12 000 g for 10 min at 4 °C and added 200 µL of MDA assay working solution. After mixing, the solution was heated at 100 °C for 15 min. The water bath was cooled to room temperature and centrifuged 1000 g at room temperature for 10 min. Two hundred microliters of supernatant was added to a new 96‐well plate, and the absorbance was measured at 532 nm using a microplate reader.

### The PMR Activity of AMDs—Cell viability and LDH release assays

Tumor cells (4T1, CT26, and EMT6) were seeded on a 48‐well plate at a density of 2 × 10^4^ cells per well and incubated for 24 h at 37 °C before the assay. The culture medium was then replaced with fresh medium containing AMDs (the ratio of AMDs to tumor cells, 5). After 6 h incubation at 37 °C, the AMDs‐containing medium was removed, and fresh culture medium was added. Irradiation (660 nm, 1.0 W cm^−2^, 5.0 min) was carried out for 6 h. The results were determined with CCK‐8 and Cytotoxicity LDH Assay Kit, and the data were processed with GraphPad Prism 8.0 (GraphPad Software, CA) to calculate cell viability curves. After pretreating 4T1 cells with Ferrostatin‐1 (2 µm, 24 h), Liproxstatin‐1 (1 µm, 24 h), Trolox (1 µm, 24 h), Z‐VAD‐FMK (5 µm, 24 h), KCl (20 mm, 24 h) or MCC950 (1 µm, 24 h), AMDs were added and then treated with light (660 nm, 0.2, 0.5, or 1.0 W cm^−2^, 5.0 min) to detect LDH release. Experiments were performed according to the protocol provided by the supplier, and all tests were repeated at least three times.

### The PMR Activity of AMDs—Transwell Assay

4T1 cells were cultured in the top wells (10^5^ cells per well), and AMDs (the ratio of AMDs to tumor cells, 5) were cultured in the bottom wells. After exposure to light (660 nm, 1.0 W cm^−2^, 5.0 min), toxicity was measured by LDH release. A transwell polycarbonate membrane cell culture insert kit (Labselect, 3.0 µm pore size) was mounted into a 24‐well cell culture plate for this study.

### Immunoblotting Analysis

Western blotting was used to detect the protein expression in tumor cells treated with AMDs and light. 4T1, EMT6, and CT26 cells (1 × 10^6^ per well) seeded in a 6‐well plate were added to AMDs (5 × 10^6^ per well) after they adhered, incubated at 37 °C for 6 h and then treated with light (660 nm, 1.0 W cm^−2,^ 5.0 min), and the protein was extracted after 6 h. Briefly, separated proteins were transferred to PVDF membranes (Merck Millipore) by SDS‐PAGE. After 10 min blocking in Protein Free Rapid Blocking Buffer (Epizyme Biotech), incubated overnight at 4 °C with diluted primary antibody (1:1000). After washing five times with TBST, it was further incubated with diluted Anti‐rabbit IgG, HRP‐linked Antibody (1:5000) for 90 min. After five washes in TBST, the membrane was immersed in Thermo Scientific SuperSignal West Pico PLUS chemiluminescent substrate and exposed using an Amersham lmageQuant 800 (Cytiva). For 4T1, EMT6, and CT26 cells, the expression of GSDMD and GSDME was detected.

### Cytokine Measurement

The release of inflammatory factors of AMDs bound 4T1 cells treated with light (660 nm, 1.0 W cm^−2^, 5 min) were detected by indicated ELISA kits. 4T1 cells, AMDs, and AMDs bound 4T1 cells were seeded in a 6‐well plate (10^6^ cells per well) incubating for 12 h. Suspensions in well plates were collected after light and centrifuged at 3000 rpm for 30 min at 4 °C to measure IL‐1β, IL‐18, HMGB1, and TNF‐α. Data were presented as mean ± s.d (*n* = 3, independent experiments).

### Systemic Inflammatory Response

On day 1, 2, 3, 4, and 5 after intravenously injection of AMDs and Ce6, blood was gained from the eyeballs of mice to detect RBC, WBC, and PLT, and serum IL‐6 concentration was detected by ELISA. In addition, the rectal temperature and body weight of mice were measured.

### Tumor Targeting, Penetration, and Biodistribution—In Vivo Tumor Penetration Study

When the tumor volume reached 150 mm^3^, the 4T1 tumor‐bearing BALB/c female mice (6 weeks old) were intravenously AMDs (5 × 10^6^ units per mouse). Twenty four hours post‐injection, tumors were harvested and washed with PBS, followed by a well‐established frozen section protocol (Leica CM3050S). The frozen sections of the tumors were analyzed and imaged with confocal microscopy (Olympus FV3000).

### Tumor Targeting, Penetration, and Biodistribution—Preparation of Fe_3_O_4_‐DHCA

Monodisperse Fe_3_O_4_ was prepared from ferric acetylacetone in oleic acid by high‐temperature thermal decomposition. Iron (III) acetylacetonate (12 mmol) and oleic acid (38 mmol) were added to benzyl ether (50 mL) and stirred magnetically under nitrogen for 30 min. The reaction mixture was slowly heated to 165 °C for 30 min, followed by reflux at 280 °C under nitrogen for another 30 min. Once the reaction was over, the black–brown mixture was cooled to room temperature. The precipitate was cleaned with ethanol three times and the precipitate under the condition of external magnet was collected. The hydrophobic monodisperse Fe_3_O_4_ was transferred to the aqueous phase by ligand exchange reaction to obtain Fe_3_O_4_‐DHCA. Fifty milligram DHCA was dissolved in 6 mL tetrahydrofuran (THF) in a three‐necked flask (25 mL). The solution was heated to 50 °C under argon. Then, 18 mg of monodisperse Fe_3_O_4_ was dispersed in 1 mL of THF and added drop by drop. After the reaction for 3 h, it was cooled to room temperature, and Fe_3_O_4_‐DHCA was precipitated by adding 500 µL NaOH (0.5 m). Precipitation was collected by centrifugation (5000 rpm min^−1^) and redispersed in water.

### Tumor Targeting, Penetration, and Biodistribution—Preparation of AMDs‐Fe_3_O_4_.AMDs‐Fe_3_O_4_ is Prepared in a Similar Way to AMDs

The Fe_3_O_4_‐DHCA (300 µg mL^−1^) was incubated with AMDs precursors (≈10^7^ cells per dish) for 2 h and then gently washed in DMEM complete medium to produce AMDs‐Fe_3_O_4_ precursors. The precursors of AMDs‐Fe_3_O_4_ were soaked in liquid nitrogen for 24 h, defrosted at 37 °C, centrifuged at 1000 rpm for 3 min, washed, and then resuspended in DMEM complete medium or PBS to produce AMDs‐Fe_3_O_4_.

### Tumor Targeting, Penetration, and Biodistribution—MPI Imaging

Tumor cells (4T1 × 10^6^ cells) in 25 µL PBS were implanted into the right flank of BALB/c female mice (6 weeks old). When the tumor volumes reached ≈150 mm^3^ (7–10 days), the mice were randomly divided into five groups (*n* = 3 in every group), then AMDs‐Fe_3_O_4_ (2 × 10^6^ units per mouse) were injected intravenously. MPI imaging was performed using an MPI scanner (Magnetic Insight Inc, MOMENTUM). The frequency of MPI was 45 kilohertz. The magnetic gradient intensity of MPI was 0–5.5 T m^−1^. During the experiment, mice were anesthetized with 2% isoflurane at 2 L min^−1^ oxygen flow. In addition, tumors and major organs (heart, liver, spleen, lungs, and kidneys) were collected at 12, 24, 48, 72, and 96 h after injection and imaged by an MPI scanner (Magnetic Insight Inc, MOMENTUM). The analysis of MPI imaging data processed by VivoQuant software 2.0.

### In Vivo Antitumor Study

All mice used were purchased from Vital River Laboratories. To construct the tumor model, 4T1 (1 × 10^6^ cells) in 25 µL PBS were implanted subcutaneously into the right flank of BALB/c female mice (6–8 weeks old). To assess the therapeutic effect of AMDs, mice were intravenously injected with AMDs (5 × 10^6^ units per mouse) or intratumoral injection of Ce6 (200 µg per mouse) on day 7, and the tumors were irradiated with a 660 nm light (1.0 W cm^−2^, 5 min) at a specified time point post‐injection. After tumor volume reached ≈1500 mm^3^, the mice were euthanized.

### In Vivo Antitumor Study—Evaluation of the Efficacy of AMDs+anti‐PD‐1 in the Treatment of Tumors

A 4T1 tumor model was established in female mice (6–8 weeks) using the same method. Mice were injected intravenously with AMDs (5 × 10^6^ units per mouse) on day 7 of tumor inoculation and tumor were treated with a 660 nm light (1.0 W cm^−2^, 5 min) on day 8, followed by five intraperitoneal injections of anti‐PD‐1 (5 mg kg^−1^, on days 9, 11, 13, 15, and 17). After tumor volume reached ≈1500 mm^3^, the mice were euthanized.

### FACS Analyses of Tumor‐Infiltrating Lymphocytes

All mice used were purchased from Charles River Laboratories. To construct the tumor model, 4T1(1 × 10^6^ cells per mouse) in 25 µL PBS were implanted into the right flanks of BALB/c female mice (6–8 weeks old). Mice were intravenously injected with AMDs (5 × 10^6^ units per mouse) on day 7, and each of the treatments was followed by 660 nm (1.0 W cm^−2^, 5 min) on day 8. The tumors were dissected from the surrounding fascia, weighed, and minced into pieces by a gentleMACS Dissociator (Miltenyi Biotec) on day 11–16. Cell clumps were removed through a 100‐µm cell strainer to obtain single‐cell suspensions. The suspension was washed twice with cell staining buffer (Biolegend), added 0.25 µg of anti‐mouse CD16/32 Antibody per 10^6^ cells in a 100 µL volume for 5–10 min on 4 °C to block, then added 0.25 µg anti‐CD45‐FITC, anti‐CD3‐APC/Cyanine7, anti‐CD4‐APC, and anti‐CD8‐PE/Cyanine7 antibodies per 10^6^ cells in a 100 µL volume for 20 min on 4 °C in the dark. The LIVE/DEAD Fixable Violet Dead Cell Stain Kit was used to determine cell viability during FACS analysis. The FOXP3 Fixation/Permeabilization Kit was used to stain intracellular FOXP3 following the manufacturer's instructions.

Moreover, tumor homogenates were collected, and orbital blood of mice was collected for coagulation and centrifugation to collect serum, ELISA measurements of IL‐1β, IL‐10, IL‐18, TGF‐β, and HMGB1 concentrations in the serum, and the tumor homogenates of mice with the indicated treatments.

### FACS Analysis of DC Cells in TDLNs

All mice used were purchased from Charles River Laboratories. To construct the tumor model, 4T1(1 × 10^6^ cells per mouse) in 25 µL PBS were implanted into the right flanks of BALB/c female mice (6–8 weeks old). Mice were intravenously injected with AMDs (5 × 10^6^ units per mouse) or intratumoral injection of AMDs (1 × 10^5^ units per mouse) on day 7, and each of the treatments was followed by 660 nm (1.0 W cm^−2^, 5 min) on day 8. For FACS of t DC cells in TDLNs, TDLNs were collected on day 13. The lymph nodes were dissected from the surrounding fascia, weighed, and minced into pieces by a gentleMACS Dissociator (Miltenyi Biotec). Cell clumps were removed through a 100‐µm cell strainer to obtain single‐cell suspensions. The suspension was centrifuged, and the cell pellets were washed twice with cell staining buffer (Biolegend), blocked 0.25 µg of TruStain FcX PLUS (anti‐mouse CD16/32, BioLegend) Antibody per 10^6^ cells in a 100 µL volume for 5–10 min on 4 °C, and anti‐CD45‐FITC, anti‐ CD11c‐PerCP/Cyanine5.5, anti‐ CD80‐APC, anti‐ CD86‐PE‐Cy7, and anti‐ MHC II‐BV605 antibodies at predetermined optimum concentrations (0.25 µg per 10^6^ cells in a 100 µL volume) were added and incubated on 4 °C for 20 min in the dark. The LIVE/DEAD Fixable Violet Dead Cell Stain Kit (L34963, Invitrogen) was used to determine cell viability during FACS analysis.

### Single‐Cell RNA Sequencing

FACS analysis of tumor‐infiltrating lymphocytes and tumor‐infiltrating lymphocytes isolated in tumor models was performed after staining with a PE‐conjugated anti‐mouse CD45 antibody (clone 30‐F11, Biolegend). CD45+ immune cells were then enriched using a BD FACS Aria III flow cytometer. Cell viability was monitored in real‐time during the preparation of single CD45+ immune cell suspensions. Ten thousand cells (≈600 single cells per microlitre) from each experimental group were barcoded and pooled using a 10x Genomics device. Samples were prepared according to the manufacturer's protocol and sequenced on an Illumina NextSeq sequencer. The Cell Ranger Analysis Pipeline (v.3.0.2) was used for sample demultiplexing, barcode processing, alignment, filtering, UMI counting, and aggregation of sequencing runs. For quality control of the single‐cell RNA‐sequencing process, cells with fewer than 300 genes detected and cells with mitochondrial‐encoding gene transcript counts exceeding 15% of total transcript counts were removed from subsequent analyses. Genes detected in less than three cells in the entire dataset were also excluded, resulting in a preliminary expression matrix of 20 609 cells. After obtaining the digital gene expression data matrix, dimensionality reduction, clustering, and differential gene expression analysis were performed using Seurat (v.3.0.0.9000).

### Statistical Analysis

All the values in the present study were presented as the mean ± SD unless otherwise indicated in the figure captions. One‐way analysis of variance was used for multiple comparisons when more than two groups were compared, and Student's *t*‐test was used for two‐group comparisons. All statistical analyses were conducted with the GraphPad Prism software package (PRISM 8.0; GraphPad Prism Software), Excel 2019. Survival curves were obtained using the Kaplan–Meier method and compared by the log‐rank test. The threshold for statistical significance was *p* < 0.05.

### Animal Ethics and General Protocols for Animal Studies

All mouse studies were conducted in accordance with the principles and procedures outlined in the Guide for the Care and Use of Laboratory Animals (Ministry of Health, China), and the protocol was approved by the Animal Experimental Ethical Inspection of Laboratory Animal Centre, Wenzhou Medical University. The tumor‐bearing mice were subjected to the indicated treatments when the tumor volume reached 100 mm^3^ (≈1 week after inoculation) or 200–300 mm^3^ (2–3 weeks after inoculation). Ethical compliance with the IACUC protocol was maintained. In none of the experiments did the size of the tumor graft surpass 2 cm in any two dimensions (according to the limits defined by the IACUC protocol), and no mouse had severe abdominal distension (≥ 10% increase in original body weight). The Tab of Animal Experimental Ethical Inspection of Laboratory Animal Centre, Wenzhou Medical University.

### Data Availability

All data supporting the findings of this study are included in the Article and its Supporting Information. The mass spetrometry proteomics data have been deposited to the ProteomeXchange Consortium with the dataset identifer PXD050063. Single‐cell RNA sequencing of tumor‐infiltrating immune cells were also deposited at the Genome Sequence Archive at the National Bioinformatics Centre/National Genome Data Centre. No. CRA014397. Please access from the following link: https://bigd.big.ac.cn/gsa/browse/CRA014397. Source Data are available with the paper.

## Conflict of Interest

The authors declare no conflict of interest.

## Author Contributions

X.H., H.Y., D.X., and T.C. contributed equally to this work. Q.W. conceived the study; H.Y. assisted by X.H. performed material synthesis, characterization, and chemical analysis; X.H. assisted by H.Y., Y.L., and D.X. performed imaging and data analysis; H.Y. and X.H. performed the pathological analysis; H.Y. assisted by D.X. performed all other experiments. D.X., M.L. provided technical assistance (FACS) and valuable suggestions. H.Y., Z.Z., X.H., and Q.W. analyzed the data. Q.W. and X.H. wrote the manuscript with input from all authors. All authors discussed the results and commented on the manuscript.

## Supporting information

Supporting Information

Supplemental Video 1

## Data Availability

The data that support the findings of this study are available in the supplementary material of this article.
