# Supplementary material for: A Bioinspired Immunostimulatory System for Inducing Powerful Antitumor Immune Function by Directly Causing Plasma Membrane Rupture
Source: Adv Sci (Weinh). 2024 Mar 14;11(20):2305934. doi: 10.1002/advs.202305934 (PMC11132027; doi:10.1002/advs.202305934)
Supplement: Supplementary file 1 — Supporting Information [file ADVS-11-2305934-s001.pdf]

ADVANCED SCIENCE
Open Access

Supporting Information

for *Adv. Sci.*, DOI 10.1002/advs.202305934

A Bioinspired Immunostimulatory System for Inducing Powerful Antitumor Immune Function by Directly Causing Plasma Membrane Rupture

*Xiaoqu Hu, Hao Yin, Danli Xie, Tanzhou Chen, Yida Li, Hanqian Zeng, Mingdong Lu and Qinyang Wang**

Supplementary information

A Bioinspired Immunostimulatory System for Inducing Powerful Antitumor Immune Function by Directly Causing Plasma Membrane Rupture

Xiaoqu Hu[2,*], Hao Yin[1,3,*], Danli Xie[3,*], Tanzhou Chen[2,*], Yida Li[1], Hanqian Zeng[2], Mingdong Lu[1] and Qinyang Wang[1,3,#]

[1] Department of Radiation and Medical Oncology, Wenzhou Key Laboratory of Basic Science and Translational Research of Radiation Oncology, Zhejiang Engineering Research Center for Innovation and Application of Intelligent Radiotherapy Technology, The Second Affiliated Hospital and Yuying Children's Hospital of Wenzhou Medical University, Wenzhou, Zhejiang, P.R. China.

[2] The First Affiliated Hospital of Wenzhou Medical University, Wenzhou Medical University, Wenzhou, Zhejiang, P.R. China.

[3] Institute for Advanced Research, Wenzhou Medical University, Wenzhou, Zhejiang, P.R. China.

* These authors contribute equally.

# Correspondence should be addressed to Q.W. (wangqy@wmu.edu.cn)

Corresponding author:

Qinyang Wang, Ph.D.
Wenzhou Medical University, Wenzhou
Higher Education Park
Zhejiang, P.R. China, 325035

Table of content

Materials 3
Singlet oxygen generation 3
The uptake of Ce6 by cells and its distribution on subcellular structures 3
Membrane proteins evaluation of AMDs 3
Cytokine measurement 4
PMR efficacy in deep tissue 5
Light controlled peroxidation in tumours 5
In vivo propidium iodide staining assay of Tumor cell membrane rupture type death 5
Side-effect analysis in a mouse tumour model 6
Reference 6

Materials

Alkynyl-Ce6 and FITC-DBCO were purchased from Xian ruixi Biological Technology. Ac4ManNAz and Chlorin e6 (Ce6) were purchased from MCE. ABDA was purchased from Macklin.

Singlet oxygen generation

ABDA traps singlet oxygen[1]. After co-treated with AMDs (~ 10^7 cells) and ABDA (20 μL, 5 mM) for 15 min, followed with irradiation (660 nm light) at a power of 1 W cm^{-2} for different times (5, 10, 15, 20, 25, 30, 35, 40, 45, and 50 min). The singlet oxygen was then quantified by detecting the absorbance of ABDA with ultraviolet spectrophotometer.

Confocal image capture and flow cytometry[2]. The PBS containing AMDs was added to the glass-bottom dish (1 x 10^6/dish), then SOSG reagent (2 μL, 5 mM) was added and incubated for 5 min. Laser irradiation (660 nm, 1.0 W cm^{-2}, 5 min) was performed before still image collection and flow cytometry.

The uptake of Ce6 by cells and its distribution on subcellular structures

Ce6 colocalizes with cell membranes. 4T1 cells were seeded in glass-bottom dishes (3 x 10^5 cells/dish) for 6 h until the cells adhered and washed twice with PBS. Alkynyl-Ce6 (40 μM) was incubated with cells for 2 h, washed twice with PBS, and then 500 μL of culture with Calcein-AM (10 μL, 1 mM) and DiIC18(3) (5 μL, 1 mM) were added to cells, stained for 30 min and washed 3 times with PBS. The whole process of staining should be protected from light. Representative images of co-localization of 4T1 cell membrane and Ce6 signal were observed and captured with an Olympus confocal microscope (Olympus FV3000). The displayed image data represents at least three randomly selected fields.

Membrane proteins evaluation of AMDs

The expression of SIRPα/SHPS1, CD47, Siglec-G, CD24, Integrin β1, Integrin α4 in the AMDs cells were analyzed by flow cytometry. Flow cytometric analysis was performed using Agilent Novocyte. ~ 10^6 units of AMDs were dispersed in 100 μL of cell staining buffer, and then 0.25 μg of the corresponding antibodies was added (anti-

SIRPα/SHPS1-purified, anti-CD47-PE, anti-Siglec-G-purified, anti-CD24-purified、anti-CD29-PE and anti-Integrin α4-purified), incubating at 4°C for 20 min in the dark, washing twice with 2 mL cell staining buffer. Then, 100 µL Anti-rabbit IgG (H+L), F(ab)2 Fragment (Alexa Fluor® 488 Conjugate) (1:1000) was added, incubating at 4°C in the dark for 20 min (Supplementary Figure 2a). The expression of proteins that capable of recognition on tumour cells was detected by western blotting and flow cytometry. Meanwhile, the expression of VCAM-1 in EMT6, 4T1 and CT26 cells was further examined by flow cytometry. ~ 10^6 cells of EMT6, 4T1 and CT26 were dispersed in 100 µL of cell staining buffer, and then 0.25 µg of the corresponding flow antibody (anti-VCAM-1-purified) was added, and incubated at 4°C for 20 minutes in the dark. Washing twice with 2 mL of cell staining buffer, add 100 µL Anti-rabbit IgG (H+L), F(ab)2 Fragment (Alexa Fluor 488 Conjugate) (1:1000), and incubate at 4°C for 20 min in the dark. The cells were washed twice with 2 mL of cell staining buffer and analyzed by flow cytometry using Agilent Novocyte.

Mass Spectrometry[4]. To evaluate membrane proteins on Raw 264.7 and AMDs (1 x 10^7 cells), primary membrane proteins were rapidly isolated using the Plasma Membrane Protein Separation and Cell Separation Kit (Minute™). Membrane proteins were identified by liquid chromatography tandem mass spectrometry analysis (Supplementary Figure 2c) and the resulting LC-MS/MS data were processed using the MaxQuant search engine (v.1.6.15.0).

Cytokine measurement

The release of inflammatory factors of Raw 264.7, precursor of AMDs and AMDs were detected by indicated ELISA kits[5]. Raw 264.7 and precursor of AMDs was seeded in a 6-well plate (10^6 cells/well) incubating for 6 hours. Suspensions in well plates were collected after light (660 nm, 1.0 W cm^{-2}, 5 min), and centrifuged at 3000 rpm for 30 min at 4°C for ELISA. Meanwhile, the AMDs was thawed from liquid nitrogen and washed three times with PBS. The sample was resuspended in 500 µL fresh medium and added to a 6-well plate (10^6 units/well) incubating for 12 hours. The suspension in the well plate was collected after light (660 nm, 1.0 W cm^{-2}, 5 min), following by 3000

rpm centrifugation for 30 minutes with the corresponding ELISA Kits to detect IL-1β, IL-6, IL-10, IL-18, TGF-β, TNF-α and HMGB1. Data are presented as mean ± s.d (n = 3, independent experiments).

PMR efficacy in deep tissue

For *in vitro* PMR in depth tissues[6], 4T1 cells was seeded on a 48-well plate (2×10^4 cells/well) and incubated for 6 h at 37 °C before the assay. The culture medium was then replaced with fresh medium containing AMDs (the ratio of AMDs to tumour cells, 5). After 6 h incubation at 37 °C, the AMDs-containing medium was removed, and fresh culture medium was added. Laser (660 nm, 1.0 W cm^{-2}, 5.0 min) in the presence of a piece of chicken breast tissue, then incubate for 6h. The results were determined with CCK-8 and Cytotoxicity LDH Assay Kit.

Light controlled peroxidation in tumours

4T1 tumour-bearing mice were intravenously injected AMDs (5×10^6 units/mouse) at day 7, followed by light (660 nm, 1.0 W cm^{-2}, 5 min) on days 8. Tumours were collected on day 9, and washed with PBS, followed by a well-established frozen sectioning protocol (Leica CM3050S). For lipid peroxidation staining, frozen tumor sections were treated with 100 μL PBS containing 10 μM BODIPY 581/591 C11 and incubated at 37℃ for 30 min. The frozen sections were washed three times with 2 mL PBS and then analyzed immediately on an Olympus confocal microscope. The imaging data shown are representative of at least three randomly selected fields.

***In vivo* propidium iodide staining assay of Tumor cell membrane rupture type death**

To enable propidium iodide to label tumor cell membrane rupture type death in vivo, 4T1 tumour-bearing mice were given intravenous propidium iodide (2.5 mg/kg) 24 hours after the last round of light treatment. Ten minutes later, the mice were killed and the tumour were collected, followed by a well-established frozen sectioning protocol (Leica CM3050S). The frozen sections were analyzed immediately on an Olympus confocal microscope.

Side-effect analysis in a mouse tumour model

For histological evaluation, tumor-bearing mice were killed after designated treatment, then kidney, lung, and liver were removed and fixed overnight with 4% formalin. The tissue specimens were dehydrated with gradient ethanol, then paraffin-embedded sections were sliced and stained with hematoxylin and eosin. Independent experiments were performed with three mice for each experimental group. The data were analyzed by two professional clinicians.

Reference

1. Liang, C. *et al.* A highly potent ruthenium(II)-sonosensitizer and sonocatalyst for in vivo sonotherapy. *Nature Communications* **12**, 5001, (2021).
2. Chen, B. *et al.* A pyroptosis nanotuner for cancer therapy. *Nature Nanotechnology* **17**, 788–798, (2022).
3. Liu, C. et al. A nanovaccine for antigen self-presentation and immunosuppression reversal as a personalized cancer immunotherapy strategy. *Nature Nanotechnology* **17**, 531-540, (2022).
4. Barayeu, U. et al. Hydropersulfides inhibit lipid peroxidation and ferroptosis by scavenging radicals. *Nature Chemical Biology*, **19**, 28–37 (2023).
5. Mao, C. et al. DHODH-mediated ferroptosis defence is a targetable vulnerability in cancer. *Nature* **593**, 586-590, (2021).
6. Chen, X., Chen, Y., Xin, H., Wan, T. & Ping, Y. Near-infrared optogenetic engineering of photothermal nanoCRISPR for programmable genome editing. *Proceedings of the National Academy of Sciences of the United States of America* **117**, 2395-2405, (2020).

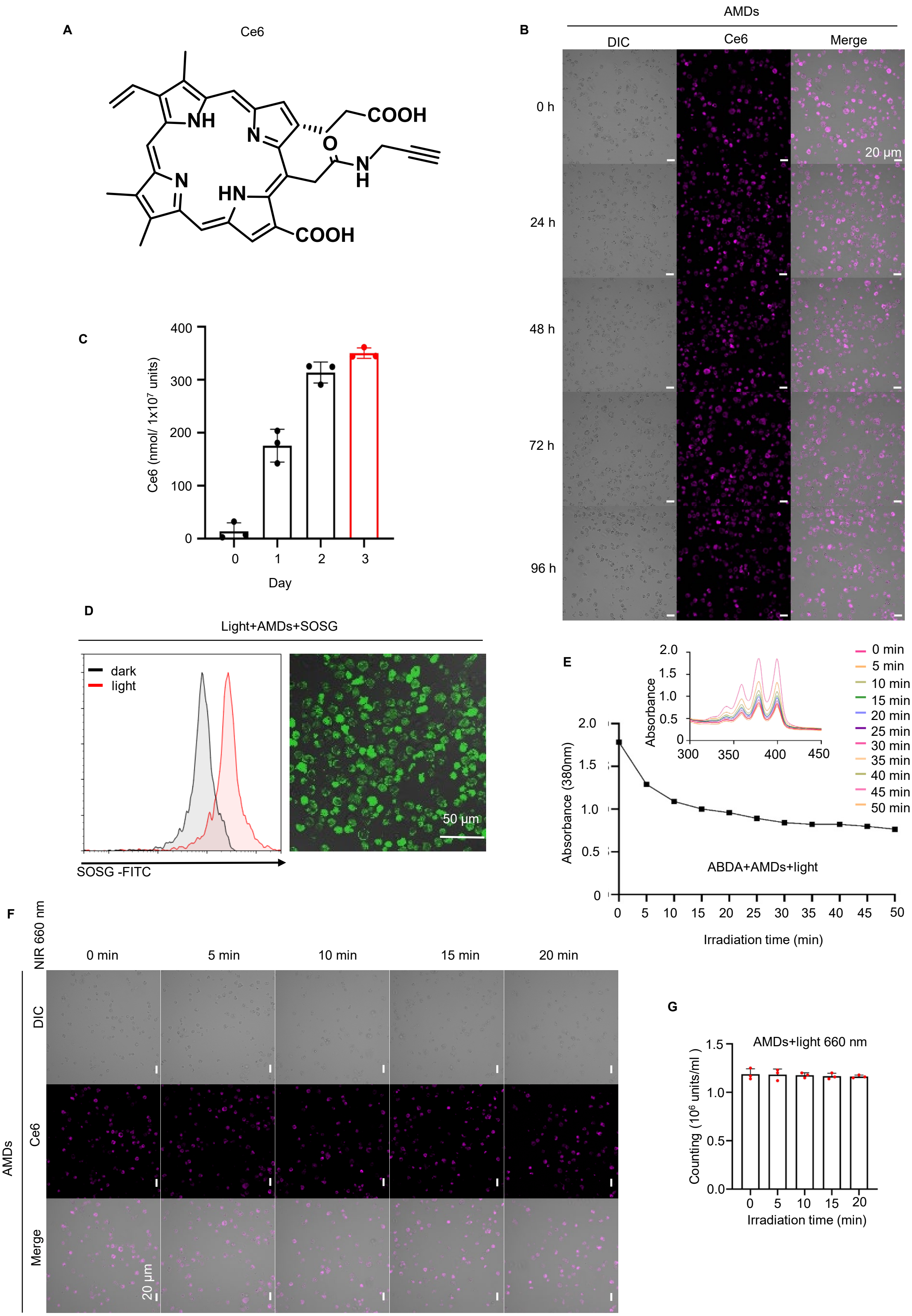
A
Ce6
COOH
NH
N
O
N
H
N
HN
COOH
B
AMDs
DIC
Ce6
Merge
0 h
24 h
48 h
72 h
96 h
20 μm
C
Ce6 (nmol/ 1x10^7 units)
400
300
200
100
0
0
1
2
3
Day
D
Light+AMDs+SOSG
dark
light
SOSG -FITC
50 μm
E
ABDA+AMDs+light
Absorbance (380nm)
Irradiation time (min)
Absorbance
0 min
5 min
10 min
15 min
20 min
25 min
30 min
35 min
40 min
45 min
50 min
F
NIR 660 nm
0 min
5 min
10 min
15 min
20 min
AMDs
DIC
Ce6
Merge
20 μm
G
AMDs+light 660 nm
Counting (10^6 units/ml)
Irradiation time (min)

Figure S1

Figure S1. Synthesis and characterization of AMDs. A, Chemical structures of Ce6. **B**, Representative fluorescence images showing the morphological stability of AMDs over a 96-hour period. **C**, Concentration of Ce6 in AMDs at different incubation times. Data are shown as mean ± s.d. (n = 3, independent experiments). **D**, Flow cytometry analysis of ROS generation and the confocal image of AMDs treated with light (660 nm, 1.0 W cm^{-2}, 5 min). Scale bars, 50 μm. **E**, The absorbance of ABDA (20 μL, 5 mM)+AMDs ($1x10^7$ units) treated with light (660 nm, 1.0 W cm^{-2}) for different times. Representative fluorescence images showing the morphological stability of AMDs over a 96-hour period. **F-G**, Confocal image (**F**) and particle size (**G**) testing of AMDs for photocorrosion and ROS corrosion resistance. 660 nm, 1.0 Wcm^{-2}, 20 min.

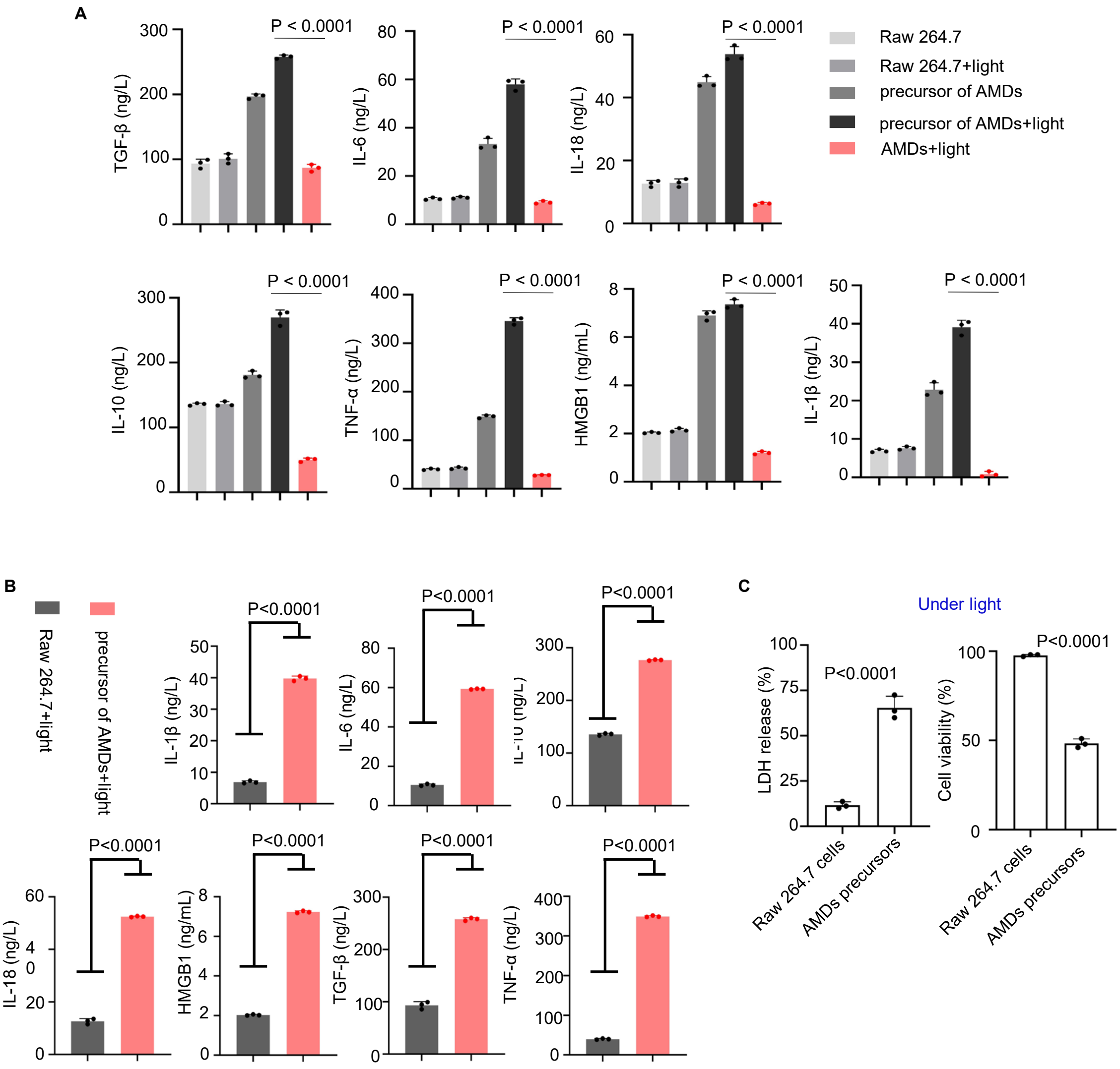

A
B
C
P < 0.0001
P<0.0001
Raw 264.7
Raw 264.7+light
precursor of AMDs
precursor of AMDs+light
AMDs+light
Raw 264.7+light
precursor of AMDs+light
TGF-β (ng/L)
IL-6 (ng/L)
IL-18 (ng/L)
IL-10 (ng/L)
TNF-α (ng/L)
HMGB1 (ng/mL)
IL-1β (ng/L)
Under light
LDH release (%)
Cell viability (%)
Raw 264.7 cells
AMDs precursors

Figure S2

Figure S2. ***In-vitro*** **safety testing of AMDs. A-B,** Secretion of TGF-β, IL-6, IL-18, IL-10, TNF-α, HMGB1 and IL-1β from Raw 264.7 cells, precursor of AMDs, AMDs with or without light (660 nm, 1.0 W cm^{-2}, 10 min) were tested by corresponding ELISA kits (**A**). Red light treatment alone does not cause inflammation in living Raw 264.7 cells. However, for Ce6 engineered living Raw 264.7 cells (precursor of AMDs), they became proinflammatory with or without light (**B**). For AMDs, the cytokines release significantly decreased compared to the control groups. **C**, Cell viability and LDH release of Raw 264.7 cells and AMDs precursors with Irradiation (660 nm, 1.0 W cm-2, 5.0 min). All data are presented as mean ± s.d. (n = 3, independent experiments, two-sided Student's *t*-test).

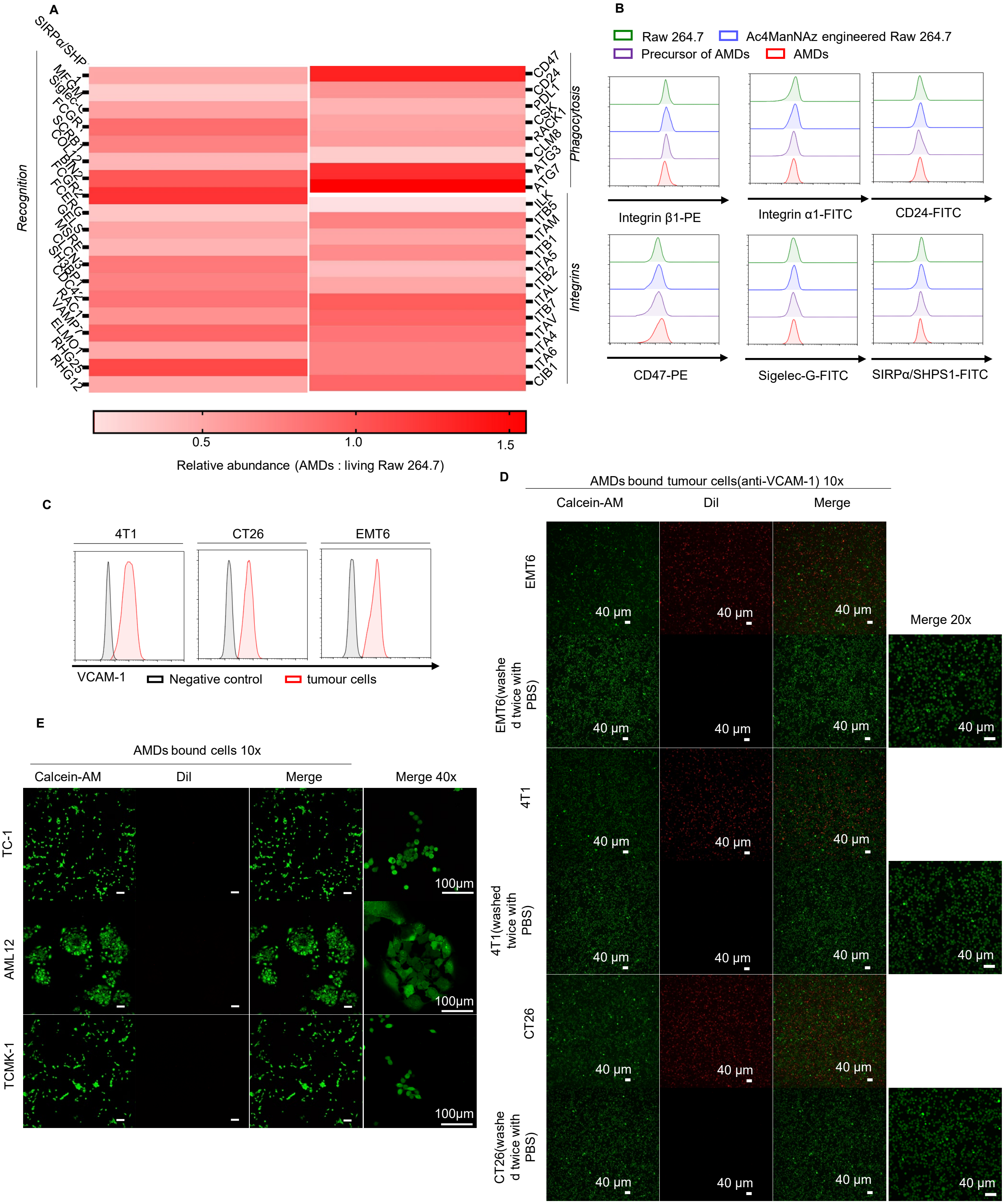

A
SIRPα/SHP
Recognition
Phagocytosis
Integrins
CD47
CD24
PDL1
CSK
RACK1
CLM8
ATG3
ATG7
ILK
ITB5
ITAM
ITB1
ITA5
ITB2
ITAL
ITB7
ITAV
ITA4
ITA6
CIB1
0.5
1.0
1.5
Relative abundance (AMDs : living Raw 264.7)
B
Raw 264.7
Ac4ManNAz engineered Raw 264.7
Precursor of AMDs
AMDs
Integrin β1-PE
Integrin α1-FITC
CD24-FITC
CD47-PE
Sigelec-G-FITC
SIRPα/SHPS1-FITC
C
4T1
CT26
EMT6
VCAM-1
Negative control
tumour cells
D
AMDs bound tumour cells(anti-VCAM-1) 10x
Calcein-AM
Dil
Merge
Merge 20x
EMT6
EMT6(washed twice with PBS)
4T1
4T1(washed twice with PBS)
CT26
CT26(washed twice with PBS)
40 μm
E
AMDs bound cells 10x
Calcein-AM
Dil
Merge
Merge 40x
TC-1
AML12
TCMK-1
100μm

Figure S3

Figure S3. Characterization of membrane proteins of AMDs and the binding. A, Relative abundance (AMDs / Raw 264.7) of recognition, negative regulation of phagocytosis and Integrin proteins. **B**, Flow cytometry of representative proteins for binding (top) and resisting to phagocytosis (bottom) in Raw 264.7 cells, Ac4ManNAz engineered Raw 264.7, Precursor of AMDs and AMDs. Raw 264.7 cells was used as a positive control. **C**, Flow cytometry of VCAM-1 expression in tumour cells (4T1, CT26 and EMT6). **D**, Confocal images between DiI-labeled-AMDs and calcein-AM-labeled cancer cells (anti-VCAM-1 pretreatment). Scale bar, 40 μm. **E,** Confocal images of the binding between DiI-labelled AMDs (red) and Calcein-AM-labelled healthy cells (green). Top, TC-1 cells. Middle, AML12 cells. Bottom, TCMK-1 cells. Scale bar, 100 μm.

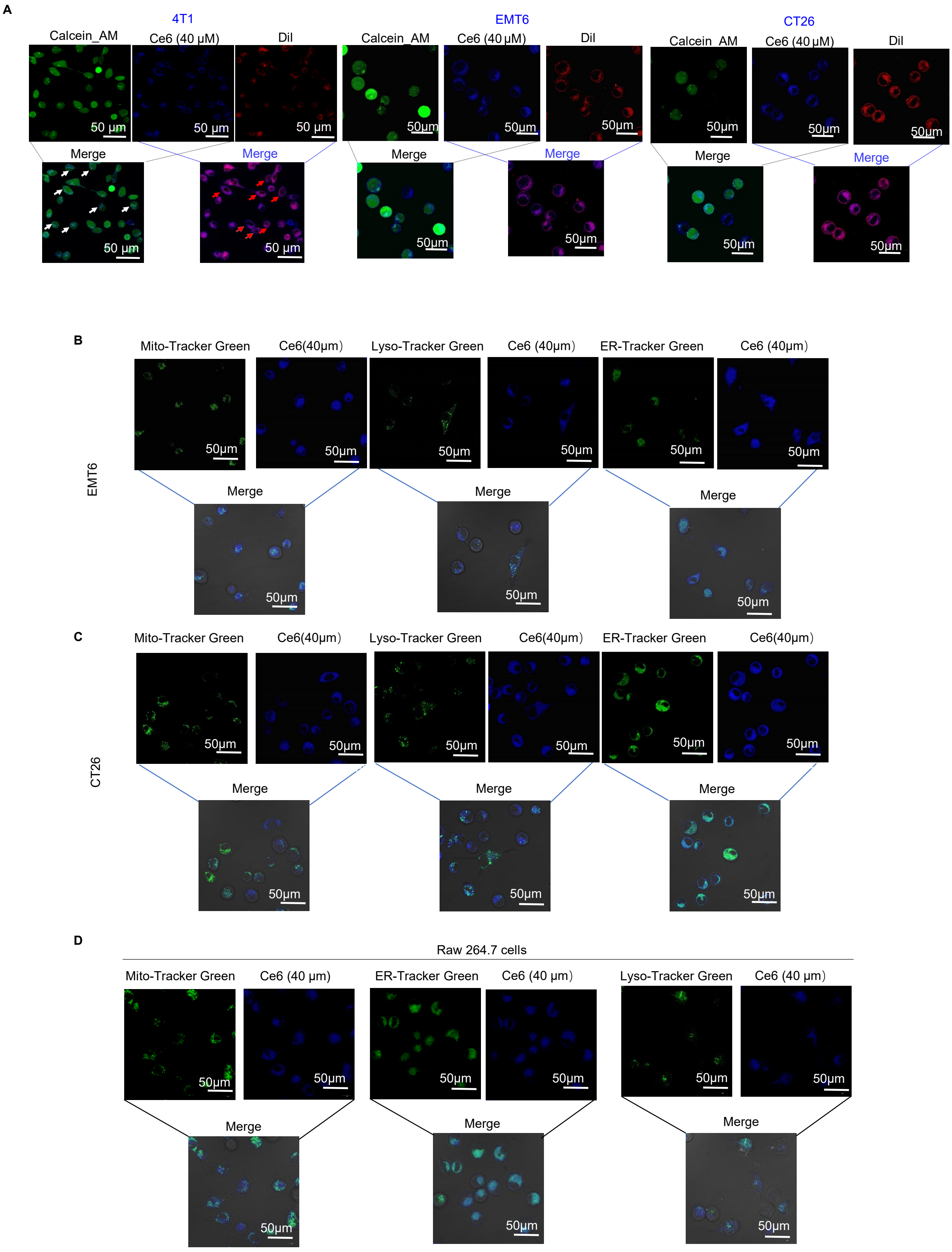
A
4T1
Calcein_AM
Ce6 (40 μM)
Dil
50 μm
Merge
EMT6
Calcein_AM
Ce6 (40 μM)
Dil
50μm
Merge
CT26
Calcein_AM
Ce6 (40 μM)
Dil
50μm
Merge
B
EMT6
Mito-Tracker Green
Ce6(40μm)
Lyso-Tracker Green
Ce6 (40μm)
ER-Tracker Green
Ce6 (40μm)
Merge
C
CT26
Mito-Tracker Green
Ce6(40μm)
Lyso-Tracker Green
Ce6(40μm)
ER-Tracker Green
Ce6(40μm)
Merge
D
Raw 264.7 cells
Mito-Tracker Green
Ce6 (40 μm)
ER-Tracker Green
Ce6 (40 μm)
Lyso-Tracker Green
Ce6 (40 μm)
Merge

Figure S4

Figure S4. Distribution of Ce6 on various membranous organelles. A, Representative confocal images of Ce6 distribution in 4T1, EMT6 or CT26 cells. Scale bars, 50 μm. The merged images showed the Ce6 distributed in the cytoplasm or plasma membrane of indicated cells. The white arrows showed the distribution of Ce6 in the cytoplasm. The red arrows showed the distribution of Ce6 in the plasma membrane. **B**, Representative confocal images of Ce6 distribution in various membranous organelles of EMT6 cells. Scale bars, 50 μm. The merged images showed the Ce6 distributed in indicated membranous organelles. **C**, Representative confocal images of Ce6 distribution in various membranous organelles of CT26 cells. Scale bars, 50 μm. The merged images showed the Ce6 distributed in indicated membranous organelles. **D**, Representative confocal images of Ce6 distribution in various membranous organelles of Raw 264.7 cells. Scale bars, 50 μm. The merged images showed the Ce6 distributed in indicated membranous organelles.

A

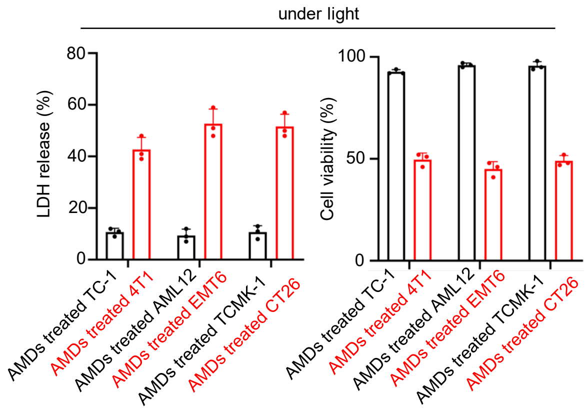

under light
LDH release (%)
80
60
40
20
0
Cell viability (%)
100
50
0
AMDs treated TC-1
AMDs treated 4T1
AMDs treated AML12
AMDs treated EMT6
AMDs treated TCMK-1
AMDs treated CT26

B

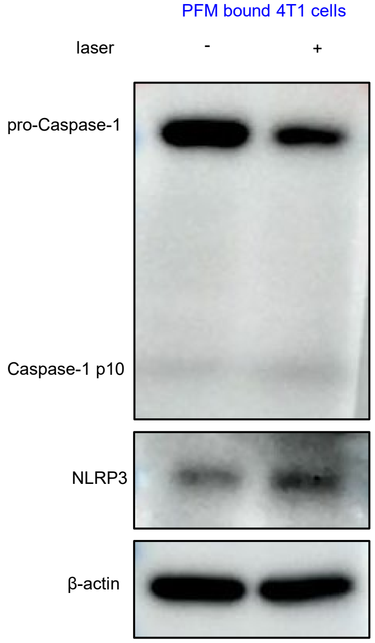

PFM bound 4T1 cells
laser
-
+
pro-Caspase-1
Caspase-1 p10
NLRP3
β-actin

Figure S5

Figure S5. Cell death analysis after light treatment for healthy cells that cannot bind to AMDs, immunoblot analysis of tumour cells. A, LDH release and Cell viability after AMDs (660 nm, 1.0 W cm^{-2}, 5.0 min) treatment of indicated tumor cells or healthy cells. The results were determined with Cytotoxicity LDH Assay Kit (DOJINDO) and CCK-8 Assay Kit. All data are presented as mean ± s.d. (n = 3, independent experiments, two-sided Student's *t*-test). **B**, Immunoblot analysis of NLRP3, caspase-1 before and after treatment of AMDs bound 4T1 cells with light.

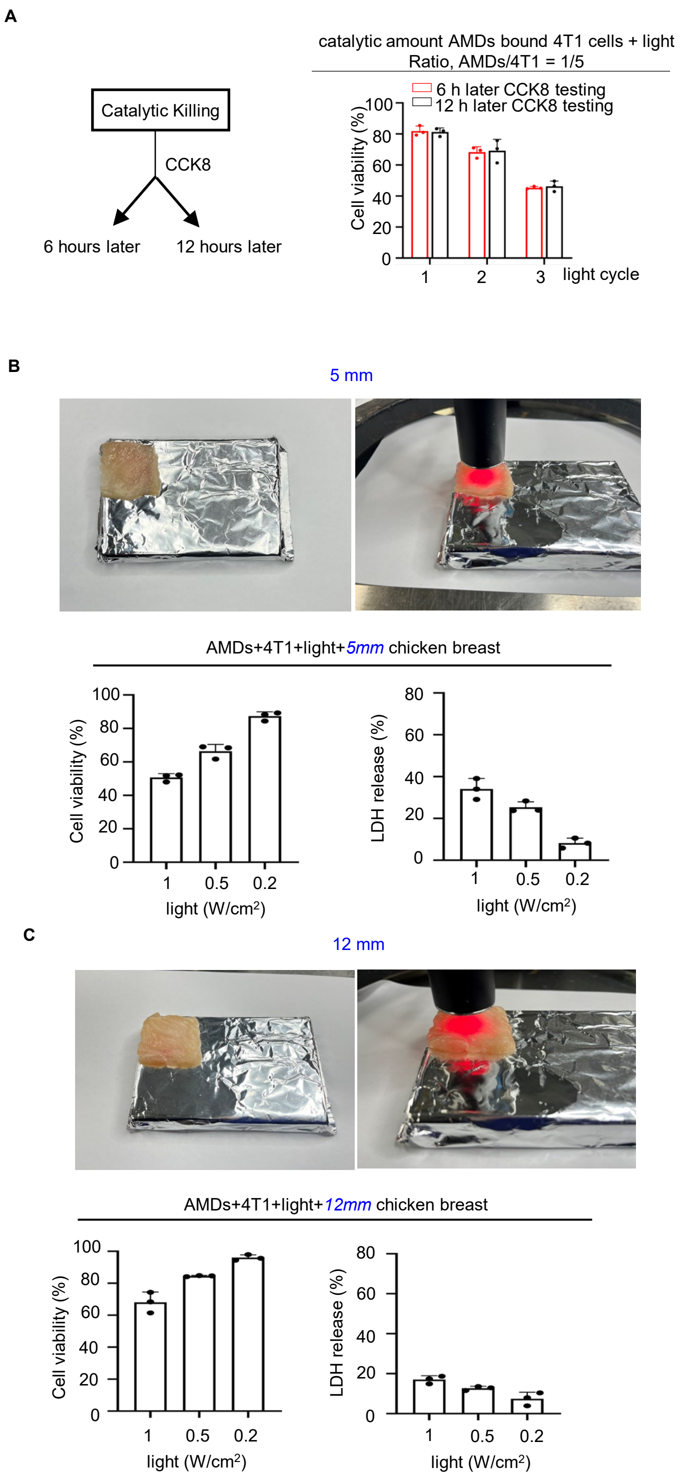
A
Catalytic Killing
CCK8
6 hours later
12 hours later
catalytic amount AMDs bound 4T1 cells + light
Ratio, AMDs/4T1 = 1/5
6 h later CCK8 testing
12 h later CCK8 testing
Cell viability (%)
light cycle
B
5 mm
AMDs+4T1+light+5mm chicken breast
Cell viability (%)
LDH release (%)
light (W/cm2)
C
12 mm
AMDs+4T1+light+12mm chicken breast
Cell viability (%)
LDH release (%)
light (W/cm2)

Figure S6

Figure S6. AMDs mediated catalytic killing in deep tissues. A, CCK8 analyses of AMDs bound 4T1 cell death at 6 and 12 hours after light treatment. AMDs/4T1 = 1/5. **B**, 5 mm chicken breast tissue. **C**, 12 mm chicken breast tissue. Illustration of cultured cells exposed to light in the presence of a piece of chicken breast tissue (Top). The CCK-8 and LDH release assays of AMDs bound 4T1 cells treated with light (660 nm, 1.0 W cm^{-2}, 10 min) in the presence of breast chicken tissue of different thicknesses (Bottom). All data are shown as mean ± s.d. (n = 3, independent experiments).

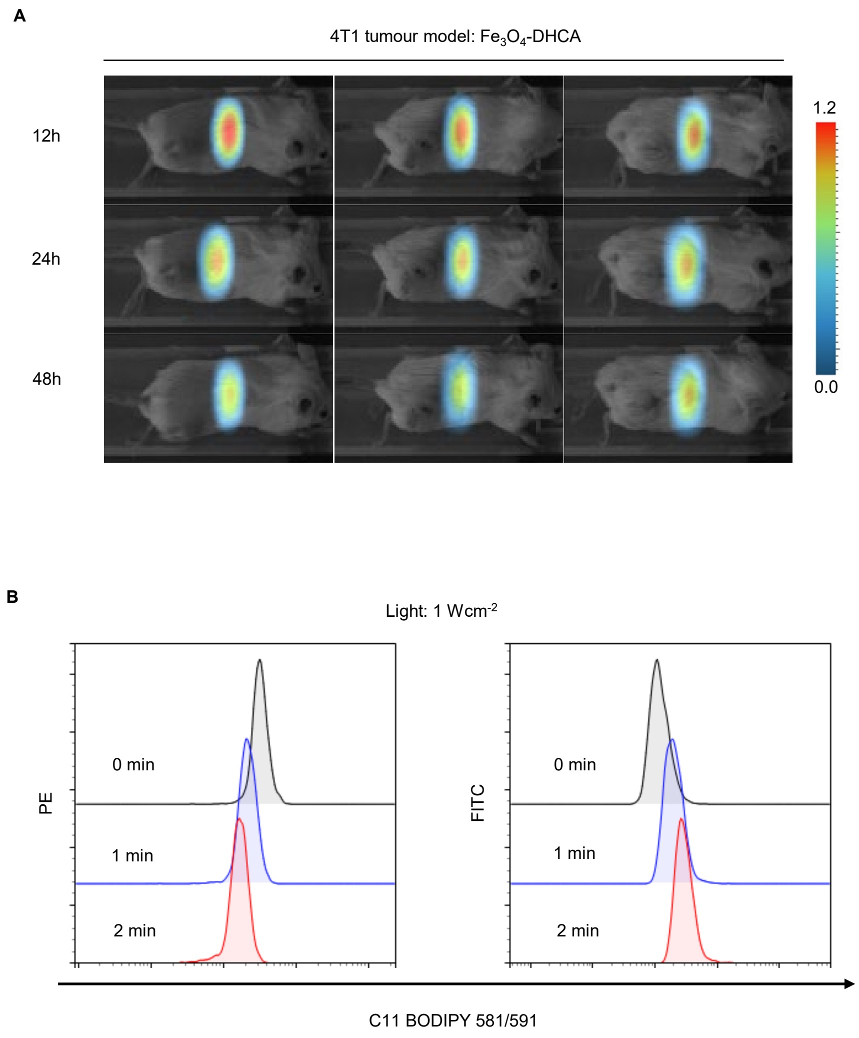
A
4T1 tumour model: Fe3O4-DHCA
12h
24h
48h
1.2
0.0
B
Light: 1 Wcm-2
PE
0 min
1 min
2 min
FITC
0 min
1 min
2 min
C11 BODIPY 581/591

Figure S7

Figure S7. Light controlled PMR in tumours. A, The MPI images of three independent 4T1 tumour bearing mice intravenously injected with Fe_3O_4-DHCA for 12, 24, 48 hours. **B,** Flow cytometry analysis of lipid peroxidation based on BODIPY® 581/591 C11 stained AMDs bound 4T1 cells treated with light (660 nm, 1 W cm^{-2}, 1 or 2 min). FITC channel, oxidized BODIPY; PE channel, non-oxidized BODIPY.

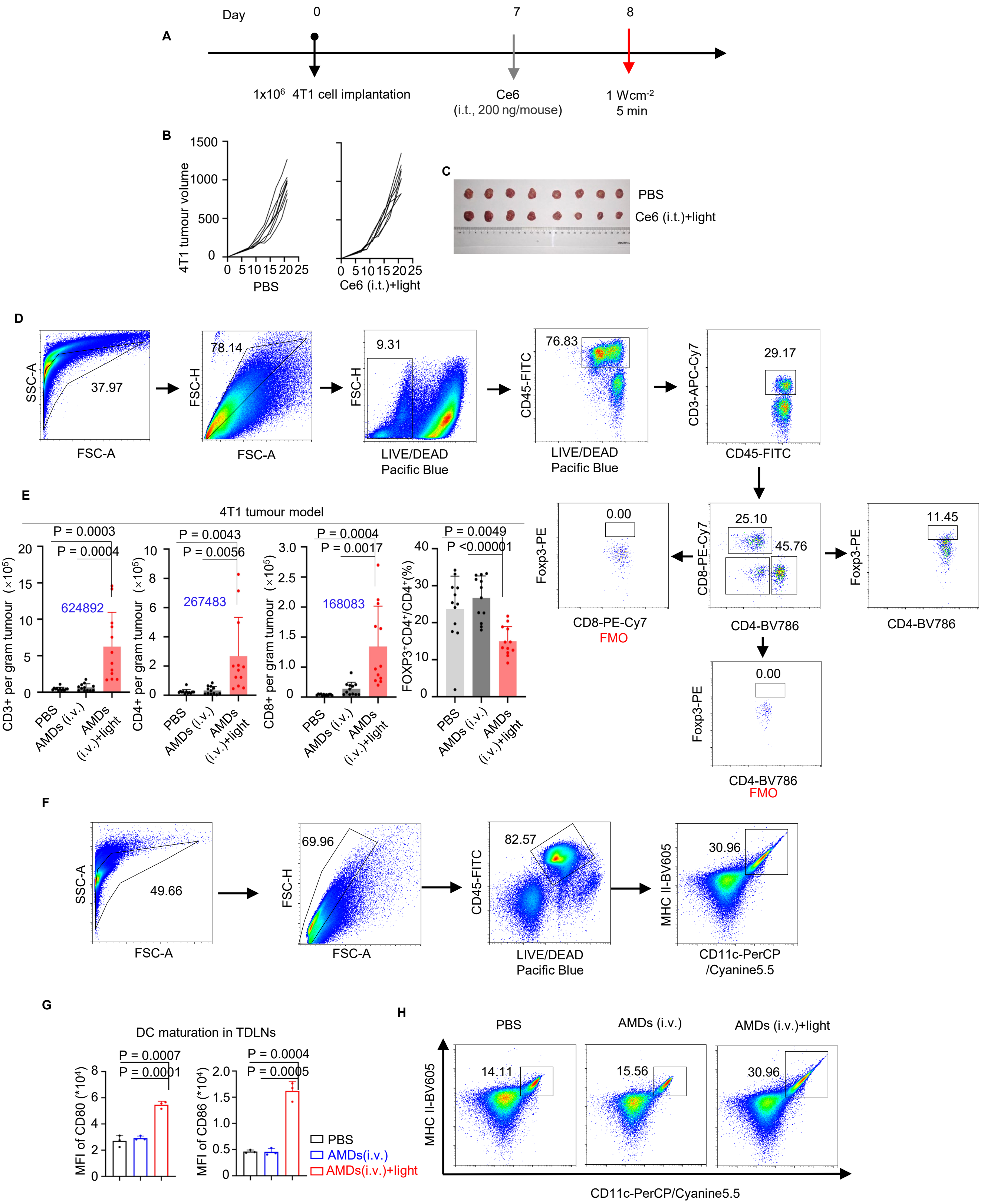

A
Day
0
7
8
1x10^6 4T1 cell implantation
Ce6
(i.t., 200 ng/mouse)
1 Wcm-2
5 min
B
4T1 tumour volume
PBS
Ce6 (i.t.)+light
C
PBS
Ce6 (i.t.)+light
D
SSC-A
FSC-A
37.97
FSC-H
78.14
9.31
LIVE/DEAD
Pacific Blue
CD45-FITC
76.83
CD3-APC-Cy7
29.17
E
4T1 tumour model
CD3+ per gram tumour (×10^5)
P = 0.0003
P = 0.0004
624892
CD4+ per gram tumour (×10^5)
P = 0.0043
P = 0.0056
267483
CD8+ per gram tumour (×10^5)
P = 0.0004
P = 0.0017
168083
FOXP3+CD4+/CD4+(%)
P = 0.0049
P <0.00001
PBS
AMDs (i.v.)
AMDs (i.v.)+light
Foxp3-PE
0.00
CD8-PE-Cy7
FMO
25.10
45.76
CD4-BV786
11.45
F
49.66
69.96
82.57
MHC II-BV605
30.96
CD11c-PerCP
/Cyanine5.5
G
DC maturation in TDLNs
MFI of CD80 (*10^4)
P = 0.0007
P = 0.0001
MFI of CD86 (*10^4)
P = 0.0004
P = 0.0005
PBS
AMDs(i.v.)
AMDs(i.v.)+light
H
PBS
AMDs (i.v.)
AMDs (i.v.)+light
14.11
15.56
30.96
CD11c-PerCP/Cyanine5.5

Figure S8

Figure S8. Analysis of enhancement of tumor immune response. A-C, The Ce6+light treatement in 4T1 tumour bearing mice. **A,** The treatment scheme. **B,** The tumour volume. **C,** The image of tumours in indicated groups. **D**, Gating strategy for 4T1-tumour-infiltrating lymphocytes including $CD3^+$, $CD4^+$, $CD8^+$ T cells or $FOXP3^+CD4^+$ regulatory T cells following the indicated treatments. **E**, Quantification of 4T1-tumour-infiltrating lymphocytes including $CD3^+$, $CD4^+$, $CD8^+$ T cells and $FOXP3^+CD4^+$ regulatory T cells from mice. n = 12 mice per group for PBS, AMDs and AMDs+light. Data are shown as mean ± s.d. (n = 12, independent experiments, two-sided Student's *t*-test). **F**, Gating strategy for DC maturation in TDLNs. **G**, DC maturation was analysed in TDLNs of the indicated groups using CD80 and CD86. n = 3 mice pre group, two-sided Student's t-test. **H**, Representative FACS analyses of DC maturation in TDLNs in indicated groups.

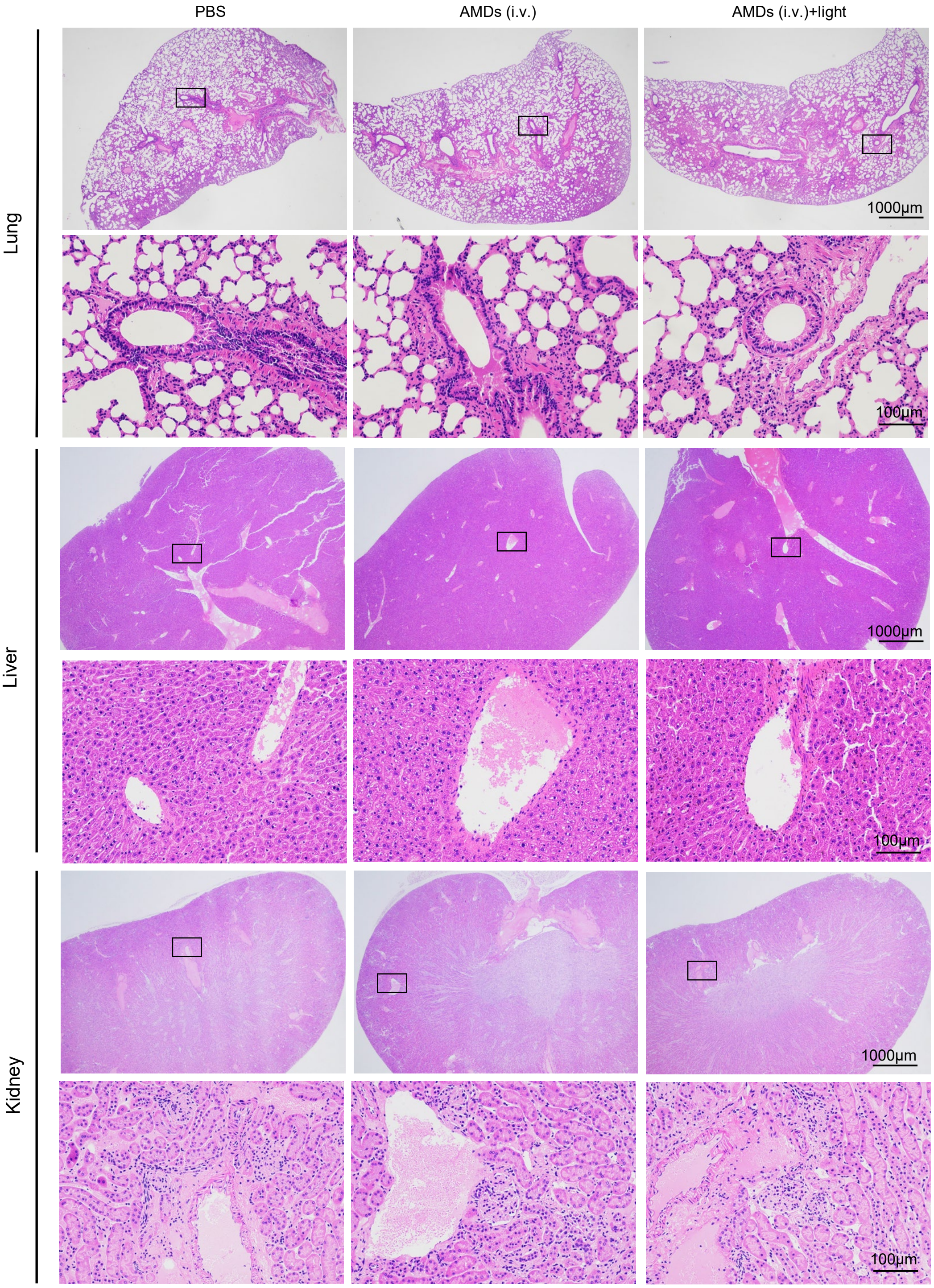
PBS
AMDs (i.v.)
AMDs (i.v.)+light
Lung
Liver
Kidney
1000μm
100μm
1000μm
100μm
1000μm
100μm

Figure S9

Figure S9. Representative haematoxylin and eosin (H & E) staining of lung, liver and kidney tissues from mice which were collected on Day 13 post-treatments. Mice treated with PBS+light, AMDs (i.v.), AMDs (i.v.)+light. Three mice per group. Scale bars, 1000 μm.

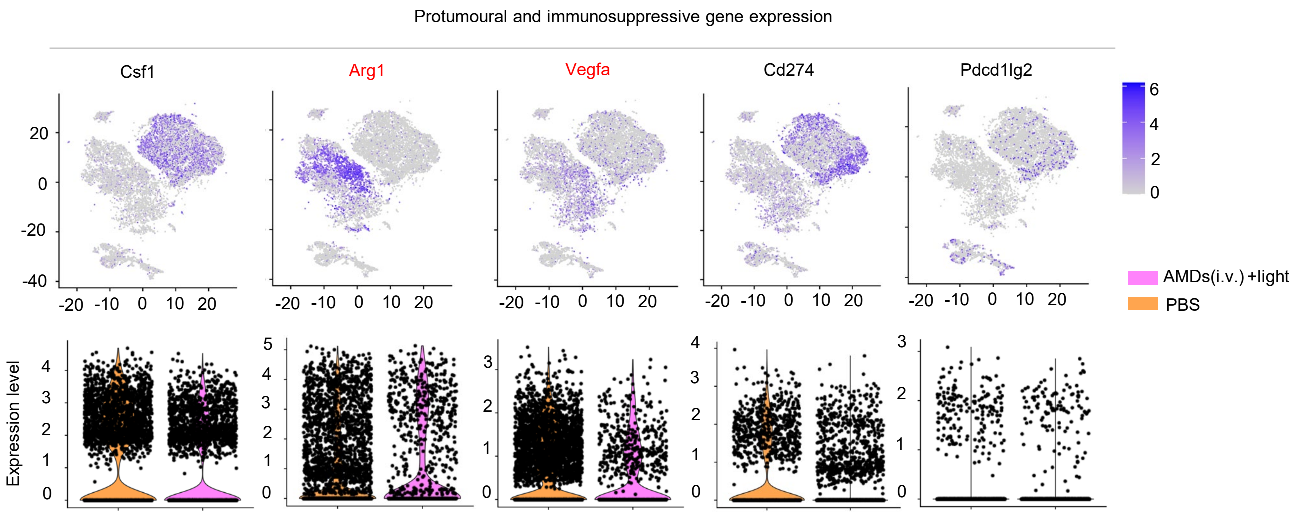

Protumoural and immunosuppressive gene expression
Csf1
Arg1
Vegfa
Cd274
Pdcd1lg2
AMDs(i.v.) +light
PBS
Expression level

Figure S10

Figure S10. The protumoural or immunosuppressive genes Csf1, Vegfa, Arg1, Cd274 (encoding PD-L1) and Pdcd1lg2 (encoding PD-L2) expression.

source data

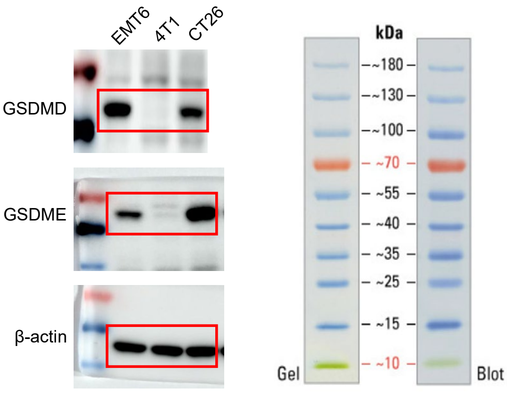

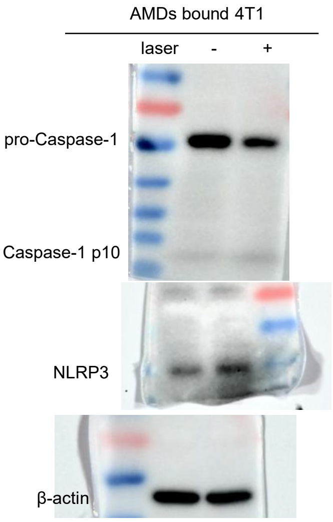

Figure S11
